# N6-methyladenosine modification of circ_0003215 suppresses the pentose phosphate pathway and malignancy of colorectal cancer through the miR-663b/DLG4/G6PD axis

**DOI:** 10.1038/s41419-022-05245-2

**Published:** 2022-09-20

**Authors:** Baoxiang Chen, Yuntian Hong, Rui Gui, Huabin Zheng, Shunhua Tian, Xiang Zhai, Xiaoyu Xie, Quanjiao Chen, Qun Qian, Xianghai Ren, Lifang Fan, Congqing Jiang

**Affiliations:** 1 Department of Colorectal and Anal Surgery, Zhongnan Hospital of Wuhan University, 430071 Wuhan, China; 2 Clinical Center of Intestinal and Colorectal Diseases of Hubei Province (Zhongnan Hospital of Wuhan University), 430071 Wuhan, China; 3 Hubei Key Laboratory of Intestinal and Colorectal Diseases (Zhongnan Hospital of Wuhan University), 430071 Wuhan, China; 4 Department of Infectious Diseases, Southwest Hospital, Third Military Medical University (Army Medical University), 400038 Chongqing, China; 5 CAS Key Laboratory of Special Pathogens and Biosafety, CAS Center for Influenza Research and Early Warning, Wuhan Institute of Virology, Chinese Academy of Sciences, 430064 Wuhan, China; 6 Department of Pathology, Zhongnan Hospital of Wuhan University, 430071 Wuhan, China

**Keywords:** Tumour biomarkers, Cancer metabolism, Colorectal cancer

## Abstract

Circular RNAs (circRNAs) are a recently discovered kind of regulatory RNAs that have emerged as critical biomarkers of various types of cancers. Metabolic reprogramming has gradually been identified as a distinct hallmark of cancer cells. The pentose phosphate pathway (PPP) plays an indispensable role in satisfying the bioenergetic and biosynthetic demands of cancer cells. However, little is known about the role of circRNAs and PPP in colorectal cancer (CRC). The novel circ_0003215 was identified at low levels in CRC and was negatively correlated with larger tumor size, higher TNM stage, and lymph node metastasis. The decreased level of circ_0003215 was resulted from the RNA degradation by the m6A reader protein YTHDF2. A series of functional assays demonstrated that circ_0003215 inhibited cell proliferation, migration, invasion, and CRC tumor metastasis in vivo and in vitro. Moreover, circ_0003215 regulated the expression of DLG4 via sponging miR-663b, thereby inducing the metabolic reprogramming in CRC. Mechanistically, DLG4 inhibited the PPP through the K48-linked ubiquitination of glucose-6-phosphate dehydrogenase (G6PD). Taken together, we have identified m6A-modified circ_0003215 as a novel regulator of metabolic glucose reprogramming that inhibited the PPP and the malignant phenotype of CRC via the miR-663b/DLG4/G6PD axis.

## Background

Colorectal cancer (CRC) is the second and third most frequently diagnosed cancer in females and males worldwide, respectively [1]. Despite significant advancements in surgical techniques, chemotherapy, immunotherapy, and molecular-targeted treatments, the overall prognosis and 5-year survival rate of CRC patients remain poor, particularly for those with distant metastatic CRC [2, 3]. Thus, it is crucial to understand the potential mechanisms underlying CRC progression, which may contribute to the enlargement of the molecular pool for the diagnosis and therapy of CRC.

CircRNAs, a newly recognized type of noncoding RNAs (ncRNAs), are generally characterized by the covalently closed loop structure without a 3′-poly-A tail or 5′-cap [4]. Unlike their linear splicing isoform, circRNAs are more stable and resistant to RNA exonuclease degradation [5]. Thus, circRNAs have been confirmed to be a valuable prognostic biomarker and promising targets in the treatment of human cancers [6–9]. N6-methyladenosine (m6A) is one of the most prevalent epigenetic modification in mRNA, microRNA (miRNA), long noncoding RNA and circRNA [10, 11]. However, the expression and biological roles of circRNAs regulated by m6A in CRC need to be further investigated.

In recent years, metabolic reprogramming has gradually been recognized as an emerging hallmark of cancer cells [12, 13]. Otto Warburg firstly observed that cancer cells presented a remarkably increased glucose consumption and preferentially produced lactate [14, 15]. The pentose phosphate pathway (PPP) is a major glucose metabolism pathway parallel to glycolysis in which ribose-5-phosphate and NADPH are generated for tumor cell nucleotide synthesis and defense against reactive oxygen species (ROS) [16]. Hence, the molecular mechanisms underlying PPP dysregulation and its relationship with the development of cancers have been gradually uncovered [17–20]. Nevertheless, the large number of circRNAs and their potential metabolic mechanisms in CRC remain to be characterized and elucidated.

In the present study, we indentified the novel circ_0003215 was significantly downregulated in CRC. Functional assays revealed that circ_0003215 inhibited the malignancy of CRC in vitro and in vivo. Importantly, circ_0003215 contains the m6A modification, which leads to the RNA degradation by m6A reader protein YTHDF2. Moreover, circ_0003215 was confirmed to inhibit PPP via DLG4-mediated K48-linked ubiquitination of G6PD protein. Overall, our results highlighted the intrinsic value of a novel circRNA as a promising early diagnostic biomarker and potential metabolic target for CRC treatment.

## Results

### Circ_0003215 is significantly downregulated in CRC

**Fig. 1:**
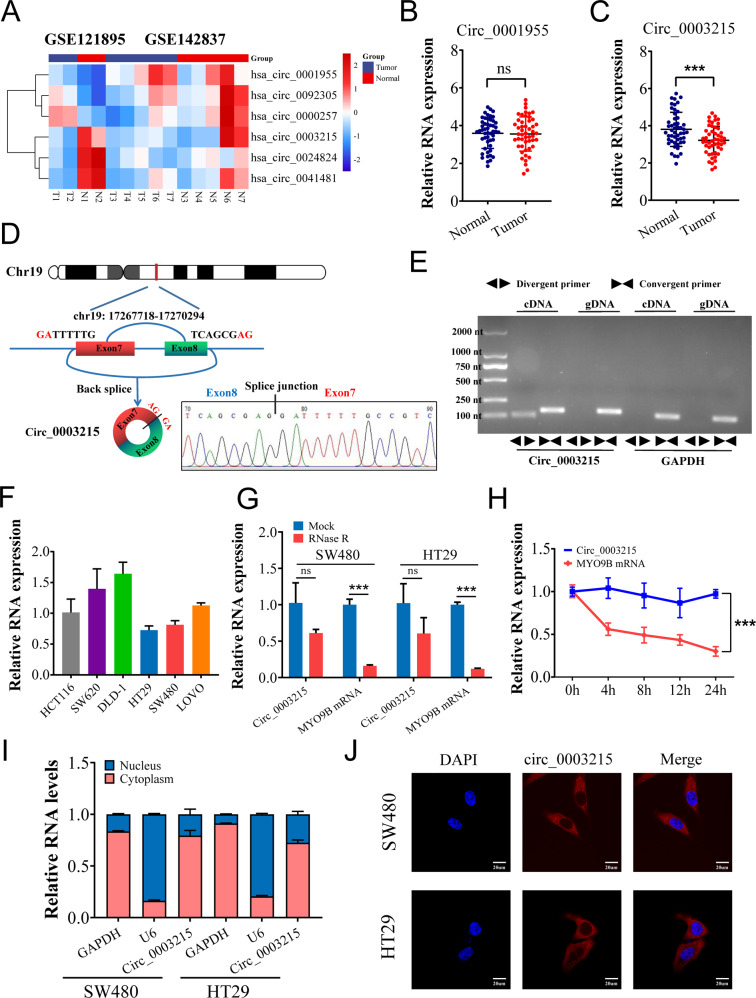
Expression profiles of circRNAs in CRC and circ_0003215 are downregulated in CRC. **A** The differentially expressed circRNAs were analyzed in GSE121895 and GSE142837(Heat map). **B**, **C** RT-qPCR analysis of circ_0001955 and circ_0003215 in 50 pairs of CRC tissues and adjacent normal tissues. **D** Schematic drawing illustrating that circ_0003215 arose from exon 7 and 8 of the MYO9B gene. The back-splicing sites (arrow) of circ_0003215 confirmed by Sanger sequencing. **E** The product of circ_0003215 and liner mRNA amplified by the convergent or divergent primers in SW480 cells by agarose gel electrophoresis. **F** Relative expression of circ_0003215 in different CRC cell lines. **G** RT-qPCR analysis for the expression of MYO9B mRNA and circ_0003215 in CRC cells after treatment of RNase R. **H** Time-course RT-qPCR analysis of circ_0003215 and its linear counterpart MYO9B in SW480 after actinomycin D (5 µg/mL) treatment. **I** RT-qPCR analysis of circ_0003215 levels in the nuclear and cytoplasmic fractions of CRC cells. **J** RNA FISH assay for the localization of circ_0003215 in SW480 and HT29 cells. *P < 0.05, **P < 0.01, ***P < 0.001.

To explore the potential circRNAs involved in the pathogenesis of CRC, the circRNA microarray (GSE121895 and GSE142837) was systematically analyzed. Several upregulated and downregulated circRNAs were visualized in volcano plots (Supplementary Fig. 1A) based on the criteria (Fold change < 2/3 or >3/2, *P* < 0.05). The intersection of six differentially expressed circRNAs was visualized using a Venn diagram (Supplementary Fig. 1B) and hierarchical clustering (Fig. 1A). Among these circRNAs, only four circRNAs (circ_0001955, circ_0024824, circ_0003215, and circ_0041481) showed consistent expression trends in both GEO datasets (Fig. 1A). Furthermore, the specific divergent primers of these four circRNAs were designed and validated using circPrimer (Supplementary Fig. 1C), and only circ_0003215 and circ_0001955 were amplified in both SW480 and HEK-293T cells (Supplementary Fig. 1D). Next, these two candidate circRNAs were verified in 50 pairs of CRC tissues, and circ_0003215 was consistently downregulated in tumor tissues according to RT-qPCR (Fig. 1B, C). The myosin IXB (MYO9B) gene encodes an F-actin–based cytoskeletal motor protein, which has been reported to suppresses the RhoA activity in lung cancer cells [21]. As is shown, circ_0003215 is located at position chr19: 17075786–17213284 and generated by back-splicing exons 7 and 8 of the MYO9B transcript (Fig. 1D). The specificity of divergent primers used to amplify circ_0003215 was confirmed using Sanger sequencing (Fig. 1D). In addition, circ_0003215 was only amplified by PCR with divergent primers from the cDNA of SW480 cells rather than gDNA, whereas the linear RNA and GAPDH had no results from divergent primers (Fig. 1E and Supplementary Fig. 1E). Then, the circ_0003215 expression was evaluated in different CRC cell lines. The cell lines (SW480 and HT29) presented the lowest expression levels and were therefore selected for follow-up analyses (Fig. 1F). Under RNase R treatment, linear MYO9B RNA levels decreased sharply, but those of circ_0003215 were unaltered (Fig. 1G). The circular structure of circ_0003215 was further validated by treating the cells with actinomycin D for different periods. The linear MYO9B RNA decreased markedly compared with circ_0003215, indicating that circ_0003215 was more stable than the linear transcript (Fig. 1H and Supplementary Fig. 1F). Furthermore, nuclear/cytoplasmic fractionation (Fig. 1I) and RNA FISH (Fig. 1J) analysis demonstrated that circ_0003215 was predominantly cytoplasmic in SW480 and HT29 cells. Analysis of the clinicopathological characteristics in our CRC cohort indicated that circ_0003215 expression levels were significantly associated with tumor size, lymph node metastasis, and TNM stage (Table 1).

**Table 1:** Correlations between the expression of circ_0003215 and various clinicopathological characteristics of 100 CRC patients.

Clinicopathological characteristic	Cases	Number of CRC patients		*P* value
		circ_0003215^Low^	circ_0003215^High^	
Age(y)	100(100%)	50(50%)	50(50%)	
≥ 60	57	33	24	0.069
< 60	43	17	26	
Gender				
Male	61	32	29	0.539
Female	39	18	21	
Tumor size (diameter, cm)				
≥ 5	32	21	11	0.032^*^
<5	68	29	39	
Tumor site				
Colon	55	24	31	0.159
Rectum	45	26	19	
Grade				
Low	84	44	40	0.275
High	16	6	10	
TNM stage				
I–II	45	16	29	0.009**
III–IV	55	34	21	
T stage				
T1-2	18	7	11	0.298
T3-4	82	43	39	
Lymph node metastasis				
pN negative (N0)	47	18	29	0.028*
pN positive (*N*+)	53	32	21	
Distant metastasis				
pM negative (M0)	91	43	48	0.162
pM positive (M1)	9	7	2	
Blood vessel invasion				
Negative	63	33	30	0.534
Positive	37	17	20	

^*^P <0.05, ^**^P <0.01.

Bold values indicates statistical significant P values.

### YTHDF2-mediated m6A modification drives the degradation of circ_0003215

**Fig. 2:**
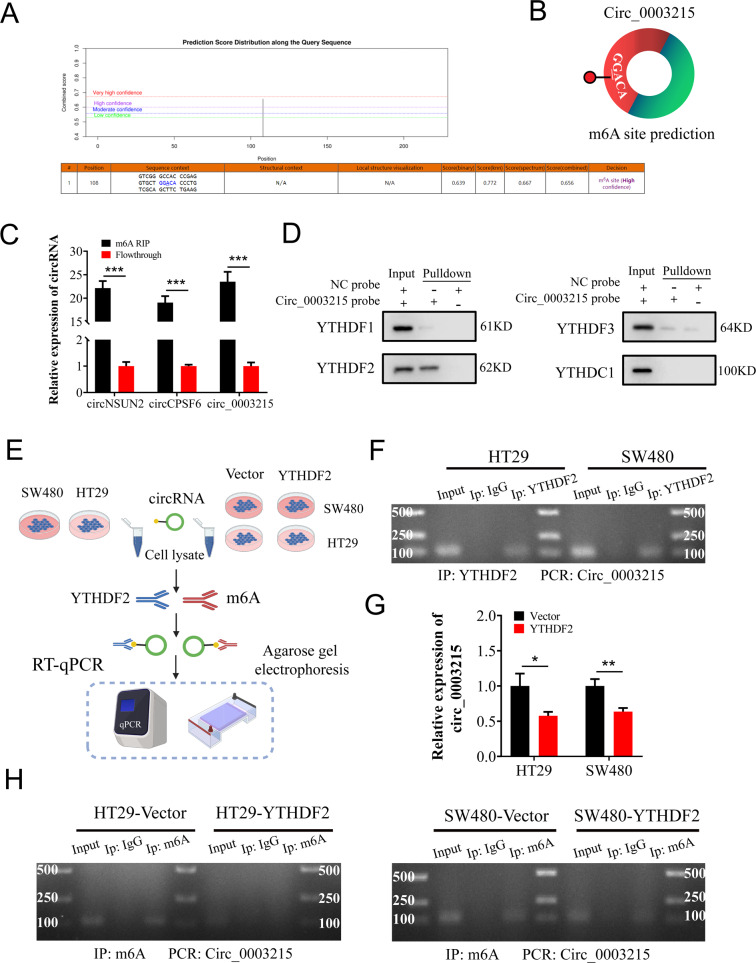
YTHDF2-mediated m6A modification drives the degradation of circ_ 0003215. **A**, **B** Predicted m6A site and motif of circ_0003215 from SRAMP. **C** MeRIP assay indicating that circ_0003215 was highly enriched in m6A precipitated fraction. **D** Western blot analysis demonstrated that only YTHDF2 protein was pull-downed by circ_ 0003215 probe. **E** Flow diagram of RIP-qPCR and agarose gel electrophoresis for circ_0003215. **F** YTHDF2 RIP assay to detect circ_0003215 in CRC cells. **G** RT-qPCR analysis for the expression of circ_0003215 after YTHDF2 overexpression in CRC cells. **H** MeRIP assays for m6A-modified circ_0003215 in HT29 (left) and SW480 (right) cells treated with empty vector or YTHDF2 overexpression plasmid.

Nevertheless, the detailed mechanisms underlying the reduction of circ_0003215 levels remain indistinct. m6A is the most widely distributed RNA modification and exerts critical roles in the metabolism of circRNAs [11]. Particularly, YTHDF2 was reported to endoribonucleolytic cleavage the m6A-modified circRNAs by YTHDF2- HRSP12- RNase P/MRP complex [22]. Of which, m6A methylation predominantly occurs at the RRACH sequence (R = A or G; H = A, C or U) [23]. We found a high confident m6A site and motif (GGACA) in circ_0003215 by using SRAMPA database (Fig. 2A, B). Moreover, two known m6A-containing cirRNAs (circNSUN2 and circCPSF6) and circ_0003215 were precipitated by m6A antibody through MeRIP analysis, validating the m6A modification of circ_0003215 (Fig. 2C). As is well known, the complex mechanisms of m6A regulation coordinated by methyltransferases (writers), demethylases (erasers), and reader proteins [24]. At present, several m6A readers (YTHDF1, YTHDF2, YTHDF3, and YTHDC1) have been confirmed to be involved in RNA degradation [22, 25–29]. Thus, RNA pull-down assay was performed and these protein were detected by western blot. The results demonstrated that the close interaction between and circ_0003215 and m6A reader protein YTHDF2 (Fig. 2D). The results of RIP assay further validated that YTHDF2 could efficiently capture circ_0003215 (Fig. 2E, F). Importantly, the expression levels of circ_0003215 and m6A levels of circ_0003215 were decreased by the overexpression of YTHDF2 (Fig. 2E and Fig. 2G-H). Taken together, these results demonstrated that YTHDF2 mediates the decay of circ_0003215 in an m6A- dependent manner.

### Circ_0003215 inhibits the proliferation, invasion, and migration of CRC in vitro

**Fig. 3:**
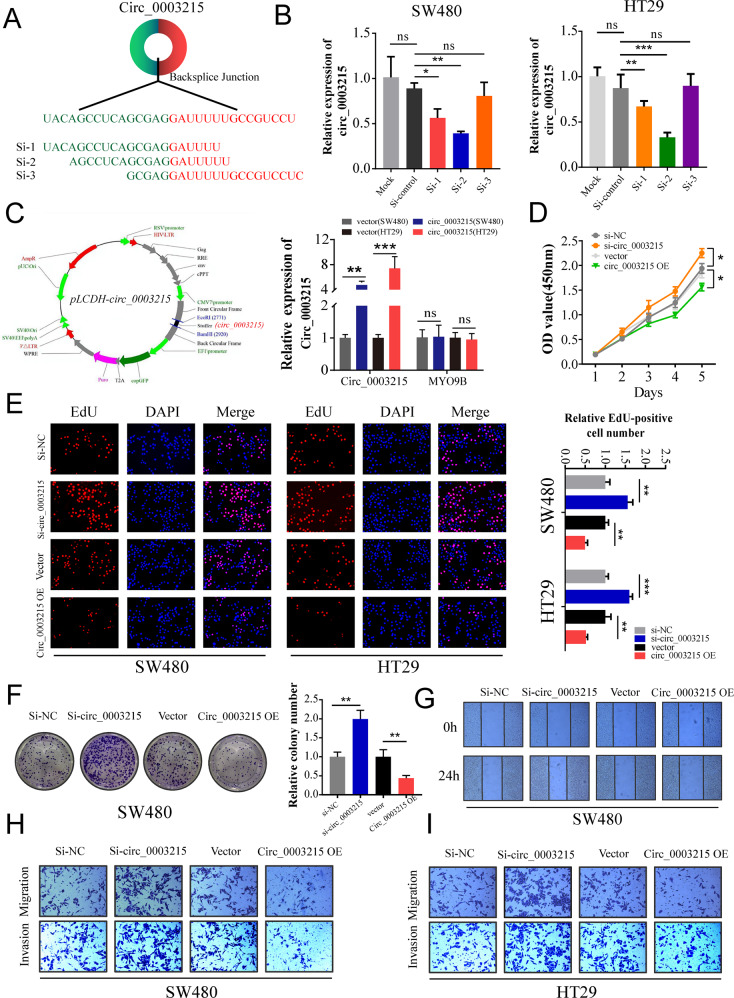
Circ_0003215 supresses CRC cell proliferation, invasion and migration. **A** Schematic illustration of three siRNAs was designed to target the back-splicing junction site of circ_0003215. **B** Knockdown efficiency of three siRNAs in SW480 and HT29 cells. Si2-circ_0003215 has the highest knockdown efficiency. **C** Schematic graph of the pLCDH-circ_0003215 plasmid constructs(left). The relative expression of circ_0003215 or MYO9B was detected by RT-qPCR after treatment with empty vector or pLCDH-circ_0003215 in CRC cells(right). **D**, **E** The cell proliferative effects of circ_0003215 were evaluated in SW480 and HT29 cells by CCK-8 and EdU assays. **F** Colony formation assay of SW480 cells transfected with control, circ_0003215 siRNA, vector or pLCDH-circ_0003215. **G** The effect of circ_0003215 on the migration of SW480 cells was detected by wound healing assay. **H**, **I** The effect of circ_0003215 on the migration and invasion of CRC cells were evaluated by transwell assay. Graph shows mean ± SD; *P < 0.05, **P < 0.01, ***P < 0.001.

We overexpressed or knocked down the circ_0003215 expression to further identify its biological functions in CRC by transfecting cells with a circ_0003215 overexpression vector or interfering RNA that specifically targets the circRNA junction site (Fig. 3A), respectively. Among the three siRNAs tested, siRNA-2 presented the most severe interference effect and was thus chosen for subsequent analyses (Fig. 3B). To increase the expression of circ_0003215, we cloned the linear sequence of the circRNA into the specific plasmid contained circular frame (Fig. 3C), which has been confirmed to efficiently facilitate the formation of circRNAs. Circ_0003215 was significantly overexpressed by transfecting with the pLCDH-circ_0003215 vector, whereas linear MYO9B mRNA was not altered (Fig. 3C). Using CCK8, EdU, and plate colony formation assays, we confirmed that the proliferation and colony formation abilities of CRC cells were enhanced after the downregulation of circ_0003215 (Fig. 3D–F and Supplementary Fig. 2A–C). Conversely, circ_ 0003215 overexpression led to the opposite effects (Fig. 3D–F and Supplementary Fig. 2A-C). Additionally, the cell migration and invasion capacities were significantly elevated when treated with siRNA, while the opposite effects were observed when circ_0003215 was overexpressed. (Fig. 3G–I and Supplementary Fig. 2D–E).

### Circ_0003215 acts as a miR-663b sponge in CRC cells

**Fig. 4:**
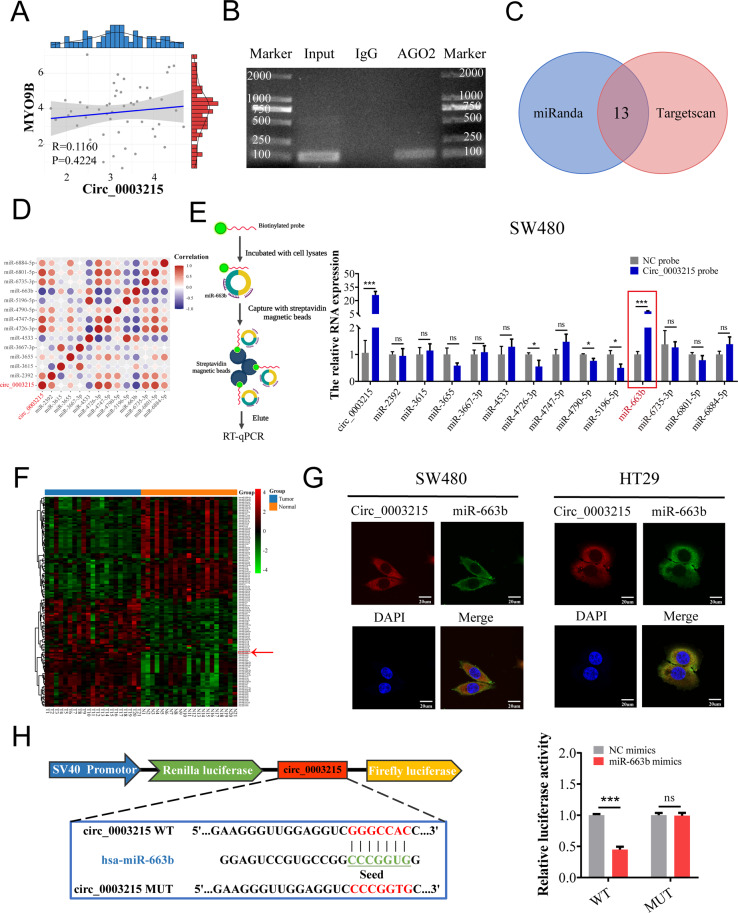
Circ_0003215 serves as an efficient miR-663b sponge in CRC. **A** Correlation analysis between the expression levels of circ_0003215 and linear MYO9B using Pearsons correlation coefficient. **B** Circ_0003215 was detected by RIP assay and agarose gel electrophoresis. **C** The potential target miRNAs of circ_ 0003215 predicted by the circBank tools. **D** The correlation matrix of circ_ 0003215 and predicated miRNAs levels measured by RT-qPCR in 10 CRC patients. (Red: positive correlation; Blue: negative correlation; circle size and color intensity are proportional to correlation coefficients.) **E** Whole-cell lysates from CRC cells were incubated with biotin-circ_0003215 or NC probe based on the RNA pull-down flow diagram(left), and the enrichment levels of circ_0003215 and predicated miRNAs were analyzed by RT-qPCR in SW480 cells(right). **F** The heatmap of miRNA sequencing data of 21 pairs of CRC tissues (GSE89143). **G** FISH asssay showed the colocalization between circ_0003215 and miR-663b in SW480 and HT29 cells. **H** The structure and the sequence of circ_0003215-WT or circ_0003215- MUT luciferase plasmid(left). The relative luciferase activities of HEK-293T co-transfected with miR-663b mimics and circ_0003215-WT compared to circ_0003215-MUT (right). *P < 0.05, **P < 0.01, ***P < 0.001.

Since our results suggested that circ_0003215 serves as a tumor suppressor in CRC, we aimed to elucidate the underlying mechanism. To examine the alternative splicing mechanism, the expression levels of linear MYO9B RNA in CRC tissues were determined. The MYO9B mRNA levels showed no significantly differential expression between CRC and adjacent normal tissues (Supplementary Fig. 3A). Similarly, no statistically significant correlation was found between circ_0003215 and linear MYO9B expression levels (Fig. 4A). Given that circ_0003215 is predominantly exhibited in the cytoplasm, it might have the biological function of competitively sponging downstream miRNAs. To assess this possibility, a RIP assay was performed. Agarose gel electrophoresis results and RT-qPCR analysis revealed that circ_0003215 was efficiently enriched using an anti-AGO2 antibody (Fig. 4B and Supplementary Fig. 3B). A total of 13 miRNAs were predicted as potential targets in circBank (Intersection of miRanda and Targetscan) (Fig. 4C). In our CRC cohort, the levels of three miRNAs (miR-663b, miR-4533, and miR-5196-5p) were negatively associated with circ_0003215 levels (Fig. 4D). Furthermore, the RNA pull-down assay revealed that the biotin-circ_0003215 probe group significantly captured more miR-663b than the NC probe in CRC cells (Fig. 4E and Supplementary Fig. 3C). Analysis of the miRNA expression microarray (Fig. 4F) of 21 paired CRC tissues demonstrated that the expression of miR-663b was elevated significantly in cancer tissues. In addition, CRC cells treated with circ_0003215-siRNA or circ_0003215 overexpression plasmid increased or decreased miR-663b expression, respectively (Supplementary Fig. 3D). Moreover, RNA FISH revealed the colocalization of circ_0003215 and miR-663b in SW480 and HT29 cells (Fig. 4G), providing further evidence of the interaction between circ_0003215 and miR-663b. The dual-luciferase reporter assay indicated that miR-663 mimics significantly reduced the relative luciferase activity of circ_003215-WT without affecting that of circ_0003215-MUT (Fig. 4H).

### miR-663b can reverse the inhibitory effects of circ_0003215 on CRC cells

**Fig. 5:**
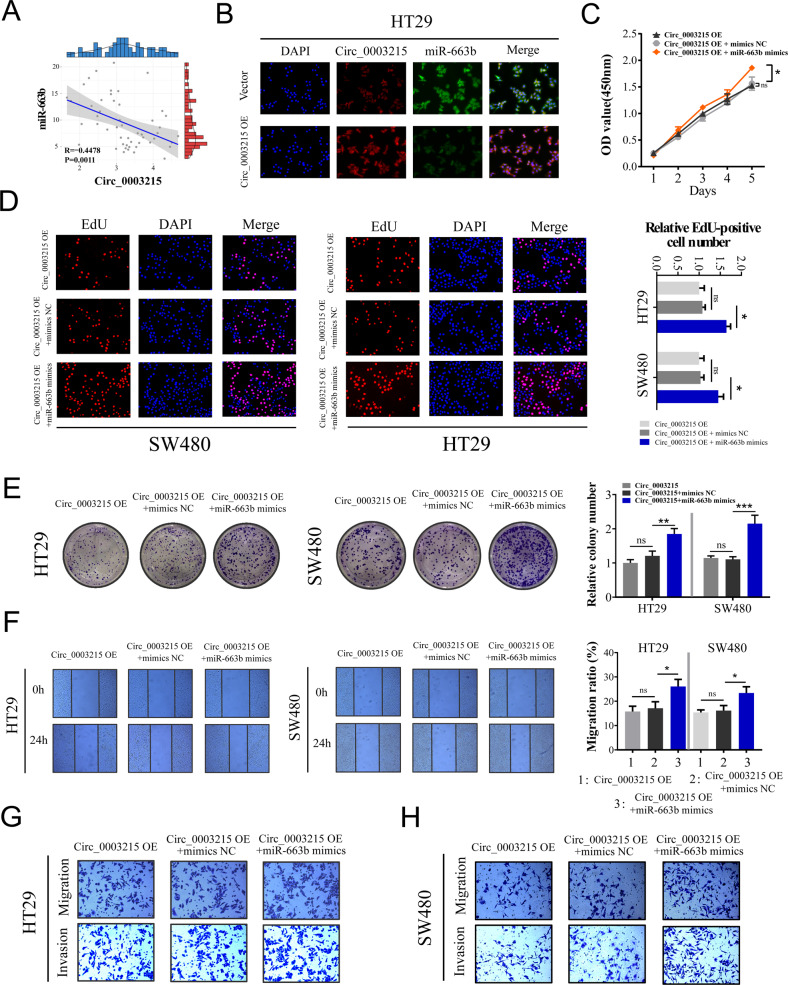
MiR-663b can reverse the inhibitive effects of circ_0003215 in CRC cells. **A** Correlation analysis between the expression levels of circ_0003215 and miR-663b expression. **B** FISH analysis of circ_0003215(red) and miR-663b(green) in HT29 cells transfected with empty vector or circ_0003215 plasmid. **C**, **D** CCK8 and EdU analysis of the proliferation capacities in HT29 or SW480 cells with diferent transfection. **E** Colony formation assay of HT29 or SW480 cells transfected with diferent transfection **F** Wound healing assay to evaluate the cell migration of HT29 cells with diferent transfection. **G**, **H** Transwell assay for the invasion and migration ability of CRC cells transfected with indicated vectors. Graph represents mean ± SD; *P < 0.05, **P < 0.01, ***P < 0.001.

To explore whether circ_0003215 functions as a tumor suppressor through miR-663b, rescue experiments were performed with circ_0003215 and miR-663b. As a result, the relative circ_0003215 levels negatively correlated with the relative expressions of miR-663b in 50 pairs of CRC samples (Fig. 5A). FISH analysis results demonstrated that transfection with circ_000325 overexpression plasmid markedly decreased miR-663b levels in HT29 and SW480 cells (Fig. 5B and Supplementary Fig. 4A). Rescue experiments revealed that miR-663b mimics could effectively reverse the inhibitory effects of circ_0003215 on the cell proliferation (Fig. 5C–E and Supplementary Fig. 4B), cell migration (Fig. 5F–H and Supplementary Fig. 4C, D) and invasion (Fig. 5G, H and Supplementary Fig. 4C, D) of HT29 and SW480. Thus, the above results confirmed that circ_0003215 plays an oncosuppressive role in CRC cells, at least in part, by sponging the downstream target miR-663b.

### Circ_0003215 regulates DLG4 levels via miR-663b sponging

**Fig. 6:**
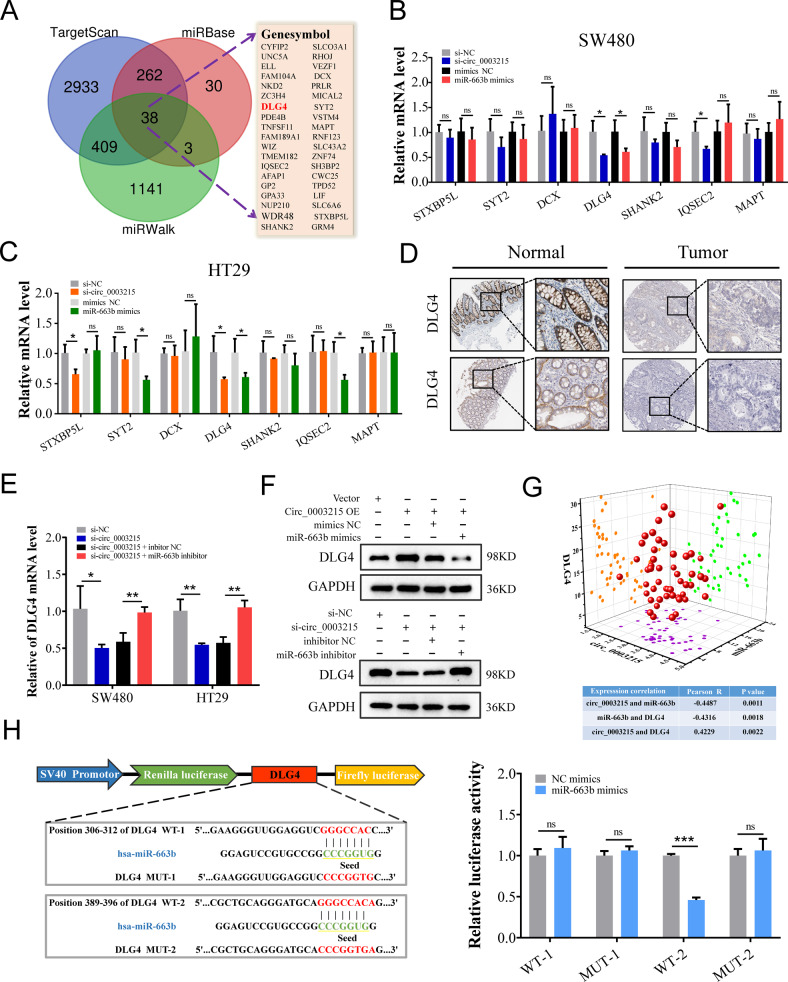
DLG4 is a direct target of circ_0003215/miR-663b axis in CRC cells. **A** The intersection of the target genes of miR-663b predicted by miRBase, miRWalk, and TargetScan. **B** The expression of potential miR-663b target genes in SW480 cells transfected with miR-663b mimics or circ_0003215-specific siRNA. **C** The expression of potential miR-663b target genes in HT29 cells transfected with miR-663b mimics or circ_0003215-specific siRNA. **D** Immunohistochemistry showed the DLG4 protein levels in CRC tissues and normal colorectal mucosa from HPA database. **E** The expresssion level of DLG4 mRNA in SW480 and HT29 cells transfected with circ_0003215 siRNA or miR-663b inhibitors. **F** Western blotting of DLG4 was detected in SW480 cells after transfecting with indicated vectors. **G** Three-dimensional scatter plot of circ_0003215, miR-663b and DLG4 in 50 paired CRC and adjacent tissues. **H** Schematic representation of the DLG4 3’-UTR WT and DLG4 3’-UTR MUT luciferase reporter vectors(left). The relative luciferase activity in HEK-293T co-transfected with miR-663b mimics or NC and the DLG4 3’-UTR WT or MUT reporter vectors (right). *P < 0.05, **P < 0.01, ***P < 0.001.

It is well known that circRNAs can competitively bind to miRNAs, thereby inhibiting the degradation of target mRNAs. Therefore, we aimed to explore the potential target genes of miR-663b indirectly regulated by circ_0003215. To address this, we predicted the target genes using three bioinformatics algorithms (TargetScan, miRBase, and miRWalk). A total of 38 intersecting genes were identified to be in common in the analysis of these three databases (Fig. 6A). Protein-protein interaction analysis of these genes using String database revealed seven hub target genes (Supplementary Fig. 5A). RT-qPCR analysis indicated that, among these genes, only DLG4 expression levels were regulated by circ_0003215 and miR-663b (Fig. 6B, C). Additionally, DLG4 was downregulated in CRC tissues in several bioinformatics databases, including GEPIA (Supplementary Fig. 5B), TIMER (Supplementary Fig. 5C), and Oncomine (Supplementary Fig. 5D). Similarly, the Human Protein Atlas immunohistochemistry results showed that DLG4 protein expression levels were lower in CRC tissues compared with those in normal colorectal tissues (Fig. 6D). Moreover, RT-qPCR and western blotting demonstrated that circ_0003215 overexpression significantly elevated the RNA and protein levels of DLG4, while miR-663b mimics attenuated this effect (Fig. 6E, F and Supplementary Fig. 5E, F). The reduction in DLG4 levels caused by si-circ_0003215 was reversed by the transfection of miR-663b inhibitor in both SW480 and HT29 cells (Fig. 6E, F and Supplementary Fig. 5E, F). Importantly, a significantly positive association between circ_0003215 expression and DLG4 levels was observed in 50 pairs of CRC tissues, whereas a negative association between miR-663b and DLG4 was observed (Fig. 6G). The predicted binding site between miR-663b and the 3′-UTR of DLG4 was further verified using dual-luciferase reporter assays (Fig. 6H). Altogether, these observations indicate that circ_0003215 regulates DLG4 expression by sponging miR-663b.

### Circ_0003215 suppresses tumorigenesis and metastasis in vivo

**Fig. 7:**
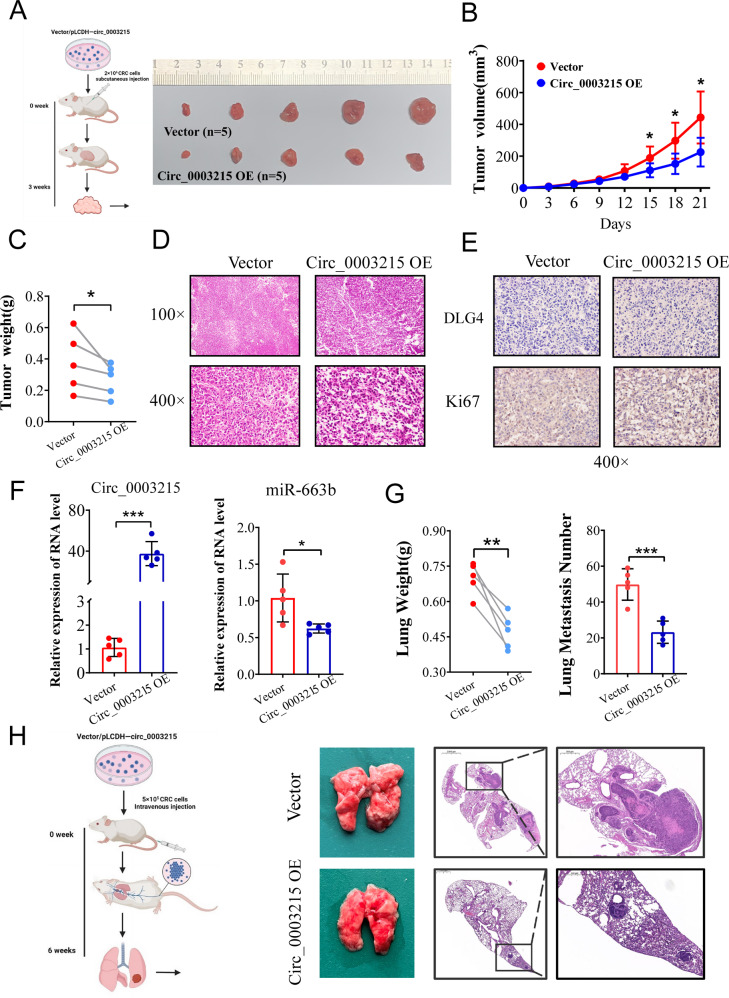
Circ_0003215 inhibits the tumor growth and metastasis of CRC in vitro. **A** Schematic flowchart of the subcutaneous tumor formation assay (left). Images of the dissected subcutaneous tumors from the tumor-bearing mice at the end of experiment (right). **B** Growth curves of xenograft tumors measured every 3 days (*n* = 5/group). **C** The final weight of the xenograft tumor was shown in the the scatter plot. **D** HE staining of the subcutaneous tumors (×100, ×400). **E** Expression of DLG4 and Ki67 in subcutaneous tumors assessed by IHC. **F** RT-qPCR analysis of relative expression of circ_0003215 and miR-663b in subcutaneous tumors. **G** Measurement of lung weight in the tail vein-lung colonization model (left). Analysis of the lung metastatic nodules under the microscope (right). **H** Schematic representation of the metastasis assay model (left). Representative images from the lungs and metastatic nodules (right). *P < 0.05, **P < 0.01, ***P < 0.001.

For in vivo assays, the subcutaneous tumor and tail vein-lung metastasis models were constructed using control CRC cells or circ_0003215 stably overexpressed cells. On the 21st day after subcutaneous tumor implantation, the mean tumor volume and weight of the circ_0003215-overexpression group were significantly lower than those of the control group (Fig. 7A–C). The tumor tissue was removed and analyzed immunohistochemically. Hematoxylin and eosin staining showed that the tumor cells grew vigorously and were arranged irregularly (Fig. 7D). Additionally, total RNA was extracted from the subcutaneous tumors and analyzed. RT-qPCR results demonstrated that circ_0003215 levels were elevated in mice injected with circ_0003215 stably overexpressed CRC cells (Fig. 7F), whereas miR-663b expression was significantly decreased (Fig. 7F). Moreover, a higher Ki67 positive cell proportion was observed in the subcutaneous tumors of the circ_0003215-overexpressing group (Fig. 7E). Additionally, DLG4 levels were remarkably upregulated in the circ_0003215- overexpressing group (Fig. 7E), further supporting the in vitro experimental results.

In the tail vein‐lung metastasis tumor model, the mean lung weight of the circ_0003215-overexpression group was significantly lower than that of the control group (Fig. 7G). Furthermore, hematoxylin and eosin staining of mice lungs revealed that the lungs of mice injected with circ_0003215-overexpressing cells had fewer metastatic nodes (Fig. 7G–H). In summary, the in vivo experiments further validated that circ_0003215 participated in the progression and metastasis of CRC.

### DLG4 regulates the PPP via G6PD in CRC

**Fig. 8:**
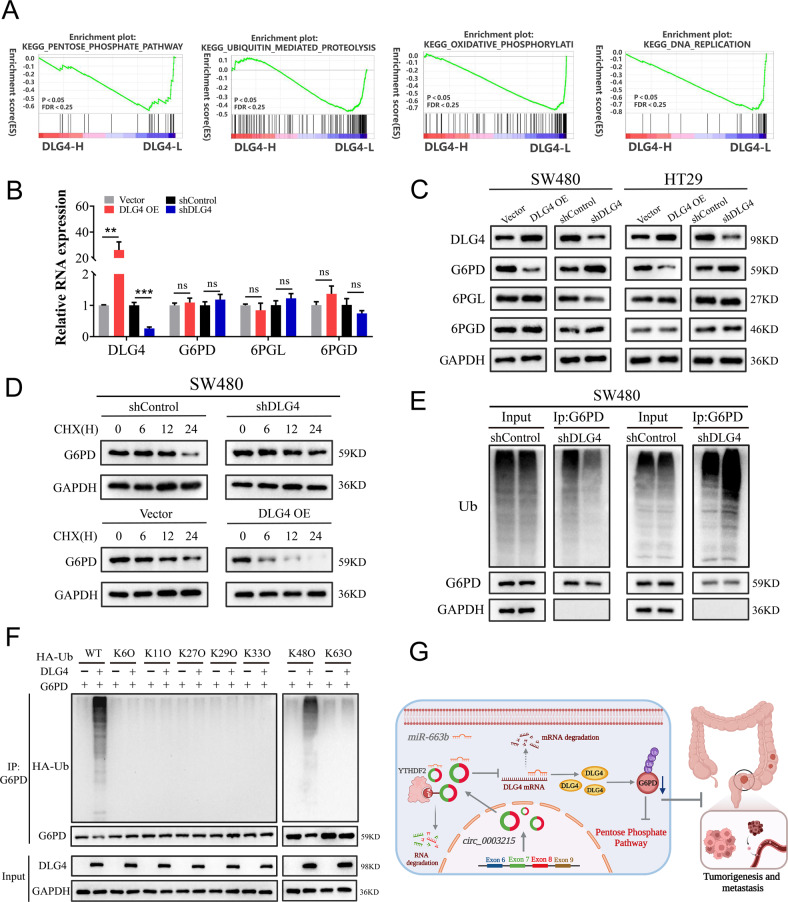
DLG4 regulates PPP through the K48-linked ubiquitination of G6PD. **A** GSEA analysis showed that low DLG4 expression associated with pentose phosphate pathway, ubiquitin-mediated proteolysis, DNA replication and oxidative phosphorylation. **B** The mRNA levels of DLG4, G6PD, 6PGL and 6PGD were detected in SW480 cells using RT-qPCR, upon overexpression or knockdown of DLG4. **C** The expression of DLG4, G6PD, 6PGL and 6PGD proteins levels were analyzed following DLG4 knockdown and DLG4 overexpression in CRC cells. **D** Degradation of G6PD was investigated using western blot analysis in a CHX chase assay in SW480 cells after DLG4 overexpression or knockdown. **E** The immunoprecipitation / western blotting assays were used to detect the ubiquitination levels of G6PD protein in SW480 cells after overexpression or knockdown of DLG4. **F** DLG4 induced K48-linked ubiquitination of G6PD in HEK-293T cells. **G** Illustration of the mechanism of circ_0003215 on promoting CRC pathogenesis and metastasis via miR-663b/DLG4/G6PD axis-mediated pentose phosphate pathway.

To assess the potential functions of DLG4 in CRC, gene set enrichment analysis (GSEA) was conducted using data from TCGA. The results indicated the enrichment of gene sets, including the PPP, ubiquitin-mediated proteolysis, oxidative phosphorylation, and DNA replication in the samples with low DLG4 expression (Fig. 8A). Moreover, according to the GEPIA database, six key enzymes of the PPP were highly expressed in CRC samples (Supplementary Fig. 6A). Altogether, these results suggested the potential role of the circ_0003215/miR-663b/DLG4 axis in regulating the PPP. To verify this hypothesis, PPP flux, NADPH/NADP^+^ ratio and ROS production were detected. The results revealed that overexpression of DLG4 reduced the oxidative PPP flux and the NADPH/NADP^+^ ratio while significantly increasing ROS production in CRC cells (Supplementary Fig. 6B). The potential mechanisms by which DLG4 influences the PPP in CRC were further investigated. The primary enzymes of oxidative PPP, including G6PD, 6PGL, and 6PGD, were detected in SW480 and HT29 cells transfected with different plasmid. As indicated, the protein levels of G6PD were remarkably decreased in SW480 and HT29 cells with DLG4 overexpression and significantly increased upon DLG4 knockdown (Fig. 8C). However, the G6PD mRNA levels remained unaltered in both cases (Fig. 8B and Supplementary Fig. 6C).

### DLG4 mediates K48-linked ubiquitination of G6PD in CRC

Accordingly, we hypothesized that DLG4 influences the PPP by regulating the G6PD protein stability. To further evaluate this hypothesis, the transfected cells were incubated with CHX for the indicated times, and then harvested and analyzed using western blotting with anti-G6PD. The results revealed that DLG4 overexpression reduced G6PD levels, whereas DLG4 knockdown increased G6PD expression in SW480 and HT29 cells (Fig. 8D and Supplementary Fig. 6D). To understand the mechanism underlying the observed effects, we explored whether G6PD protein stability was regulated by DLG4-mediated ubiquitination. Co-immunoprecipitation and western blotting demonstrated that DLG4 knockdown significantly reduced the protein ubiquitination levels of G6PD compared with those in the control group, while DLG4 overexpression promoted G6PD ubiquitination (Fig. 8E and Supplementary Fig. 6E). To further investigate the type of DLG4-mediated G6PD ubiquitination, HEK-293T cells were transfected with WT or mutant ubiquitin plasmids. In vivo ubiquitination assays suggested that DLG4 mediates K48-linked G6PD ubiquitination (Fig. 8F). Collectively, our results demonstrated that DLG4 regulated K48-linked G6PD ubiquitination, consequently influencing the PPP in CRC (Fig. 8G).

## Discussion

CRC is a highly heterogeneous disease due to the accumulation of well-characterized genetic and epigenetic alterations [30]. Notably, circRNAs have been confirmed to be expressed aberrantly in blood, exosomes and tumor tissues in the development of tumorigenesis, metastasis, and chemoradiation resistance of gastrointestinal malignancies [6–9, 31, 32]. However, the clinicopathological effects and molecular mechanisms of circRNAs in CRC still need further investigation. In the present study, we identified a novel circ_0003215, which was found to be commonly downregulated in CRC tissues. Correlation analysis revealed that a lower level of circ_0003215 is positively associated with tumor size, TNM stage, and CRC lymph node metastasis, indicating its tumor-suppressive effects. Based on these findings, we aimed to evaluate the biological effect of circ_0003215. A series of functional assays indicated that circ_0003215 suppressed the malignant traits of CRC. m6A is acknowledged as one of the most prevalent and abundant methylation modifications of RNA in eukaryotes. Recent study indicated that m6A methylation was involved in diverse biological process of many circRNAs, such as RNA stability, translation and degradation among other processes. Chen et al. found m6A-modified circCPSF6 meditated by ALKBH5 and YTHDF2 drived the malignant progression of hepatocellular carcinoma through the activation of YAP1 [33]. In addition, another study revealed modification of circNSUN2 promoted colorectal liver metastasis through facilitating cytoplasmic export and stabilizing HMGA2 [34]. Our study provides and extents further clues on the biological roles of m6A- modified circRNAs in CRC.

To date, several mechanisms by which circRNAs regulate the malignant biological behavior have been suggested and widely studied. Nuclear retained circRNAs can regulate the expression of their host gene through interacting with RNA binding proteins or alternative splicing [34]. In addition, circRNAs contain open reading frames that have been shown to be translated into the encoded proteins to exert their biological function [35]. Furthermore, it has been widely demonstrated that circRNAs can competitively bind to miRNAs, acting as “molecular sponges,” thereby attenuating the inhibitory effect of miRNAs on the expression of target genes [36]. To function as endogenous miRNA sponges, circRNAs need to present several features, including 1) being back-spliced from the exonic region of known protein-coding genes; 2) mainly located in the cytoplasm; 3) acting as potential binding sites for miRNA sponges [37–39]. Circ_0003215 is generated from the back-splicing of exons 7 and 8 of the MYO9B gene on chromosome 19. In addition, nucleoplasmic separation and RNA FISH demonstrated that circ_0003215 was predominantly exhibited in the cytoplasm of CRC cells. The potential function of circ_0003215 as a ‘miRNA sponge’ was verified using a RIP experiment with an antibody against AGO2 [40]. Subsequently, RNA pull-down, FISH colocalization, and a dual-luciferase reporter assay further suggested miR-663b as a downstream target of circ_0003215 in CRC. Besides, miR-663b has been considered a critical biomarker with the highest variable importance scores in both colon and rectal cancer datasets through the analyses of 1,894 carcinoma tissues and 1,599 normal mucosal tissues from a population-based CRC study [41]. In our CRC cohort, miR-663b expression was significantly negatively associated with circ_0003215 levels and positively correlated with DLG4 expression. Furthermore, the specific binding sequence of DLG4 and miR‐663b was validated using a dual-luciferase reporter assay. In conclusion, our study revealed that the novel circ_0003215/miR-663b/ DLG4 axis is a key regulator of CRC pathogenesis.

Recently, circRNAs-induced metabolic reprogramming of cancer cells have attracted increasing attention. For example, circMAT2B has been confirmed to promote glucose metabolism and the malignant progression of hepatocellular carcinoma under hypoxic stress through the miR-338-3p/PKM2 axis [42]. Zhou et al. found that knockdown of circMBOAT2 inhibited glutamine catabolism and tumor development in pancreatic cancer via the miR-433-3p/GOT1 axis [43]. In addition, circRNF13 suppressed glycolysis, proliferation, and metastasis of nasopharyngeal carcinoma by the SUMOylation and ubiquitination degradation of GLUT1 and AMPK-mTOR pathway [44]. However, studies on the roles of circRNAs in the PPP are scarce, especially those focusing on CRC. By determining the oxidative PPP flux, NADPH/DADP^+^ ratio, and ROS content, we found that DLG4 could regulate the PPP through altering the protein level of G6PD but not its mRNA levels. Our experimental results uncovered that DLG4 could inhibit the PPP in CRC cells by promoting K48-linked ubiquitination of G6PD. Nevertheless, the exact mechanism by which E3 ligase is responsible for this ubiquitination is unclear, and further investigations are needed.

In summary, we demonstrated the tumor-suppressing function of circ_0003215 in regulating the PPP via the circ_0003215/miR-663b/DLG4/G6PD axis. The m6A reader protein YTHDF2 promotes the RNA degradation of circ_0003215. Thus, circ_0003215 may act as a promising diagnostic marker and offer a potential basis for developing metabolic therapeutic targets for CRC in the future.

## Methods

### Microarray and RNA sequencing data

Two circRNA expression profiles (GSE121895 and GSE142837) and a miRNA expression profile (GSE115513) were downloaded from the Gene Expression Omnibus (GEO) database. The CRC RNA sequencing data (COAD, READ) were obtained from The Cancer Genome Atlas (TCGA) database. The “limma” package in R software was used to explore the significantly differentially expressed circRNAs or miRNAs.

### Tissue specimens and ethical approval

One hundred pairs of fresh human CRC specimens and their adjacent normal tissues were obtained from Wuhan University’s Zhongnan Hospital. The study participants provided their informed consent before the study began. The clinical information and pathological features of these patients were collected and are presented in Supplementary Table S1.

### Cell culture

The CRC cell lines (SW620, HCT116, SW480, DLD-1, LOVO, and HT29) and the human embryonic kidney epithelial cell line (HEK-293T) were purchased from the American Type Culture Collection (Manassas, VA, USA). HCT-116, DLD-1, HT29, and LOVO were grown in RPMI-1640 medium (Gibco; Thermo Fisher Scientific, Waltham, MA, USA) with 10% fetal bovine serum (FBS) (HyClone Laboratories Inc, Logan, UT, USA). SW480, SW620, and HEK-293T were cultured in Dulbecco’s Modified Eagle Medium (DMEM) (Gibco; Thermo Fisher Scientific) containing 10% FBS.

### RNA extraction and reverse transcription quantitative PCR (RT-qPCR)

Total RNA from cells or CRC tissues was extracted using TRIzol reagent (Invitrogen, Waltham, MA, USA). cDNA was synthesized with random primers using HiScript II Q RT SuperMix (Vazyme, Nanjing, China) or miRNA stem-loop RT primers using a miRNA 1st Strand cDNA Synthesis Kit (Vazyme). Subsequently, RT-qPCR was performed using ChamQ Universal SYBR qPCR Master Mix (Vazyme). The primer sequences are listed in Supplementary Table S2 and were synthesized by Tsingke (Nanjing, China).

### RNase R treatment assay and actinomycin D assay

For the RNase R degradation assay, total RNA (2 µg) was administrated with 3 U/µg RNase R (Epicenter). For the actinomycin D assay, SW480 and HT29 cells were cultured with 5 mg/L actinomycin D (Sigma-Aldrich, St. Louis, MO, USA) and incubated for indicated periods. Thereafter, RNA was extracted at each time point and subjected to RT-qPCR.

### Western blot

The CRC tissue samples or cells were lysed and denatured in loading buffer. Subsequently, the total proteins were resolved on sodium dodecyl sulfate-polyacrylamide gel electrophoresis (SDS-PAGE) gel and then transferred onto polyvinylidene difluoride (PVDF) membranes. After blocking in 5% non-fat milk, the PVDF membranes were reacted with primary antibodies overnight and then hybridized with the corresponding secondary antibodies. Finally, the protein bands were visualized by chemiluminescence using an ECL system. All primary antibodies are listed in Supplementary Table S3.

### Fluorescence in situ hybridization (FISH)

Specific probes for miR-663b and circ_0003215 were synthesized by GenePharma (Shanghai, China) and RiboBio (Guangzhou, China). All probe sequences are listed in Supplementary Table S4. Briefly, SW480 and HT29 cells were seeded in confocal dishes (Nest Scientific Inc, Woodbridge, NJ, USA), washed, fixed, and then permeabilized with 0.1% Triton X-100. For probe detection, CRC cells were pre-hybridized for 40 min and subsequently hybridized with the specific probe mix overnight at 37 °C. Thereafter, the CRC cells were stained with DAPI at 37 °C for 30 min after washing with a hybridization solution. Finally, the fluorescence images were acquired using a Dragonfly 200 High-Speed Confocal Microscope (Andor Technology Ltd, Belfast, UK).

### miRNAs, siRNAs, plasmid, and transfection

MiR-663b mimics, inhibitors, and small interfering RNAs (siRNAs) targeting circ_0003215 were synthesized by RiboBio. The pLCDH-circ_0003215 overexpression vector was constructed by cloning the cDNA of circ_0003215 into the specific pLCDH-circ expression vector. The short hairpin RNA (shRNA) of DLG4 was constructed by cloning the DNA sequence targeting DLG4 into the pLKO.1 plasmid. All the sequences are listed in Supplementary Table S4.

### Nuclear and cytoplasmic extraction

Cytoplasmic and nuclear fractions of CRC cells were separated using a Cytoplasmic and Nuclear RNA Purification kit (Norgen Biotek Corp, Thorold, ON, Canada). Briefly, CRC cells were harvested and lysed on ice for 15 min. After centrifugation, the nuclear and cytoplasmic fractions were collected separately. Afterward, the isolated RNAs in the nucleus and cytoplasm were extracted using TRIzol and subjected to RT-qPCR.

### Cell counting kit-8 (CCK-8) assay

Cell proliferative ability was determined using a CCK-8 assay kit (Beyotime Biotech, Beijing, China). Briefly, CRC cells were seeded in a 96-well plate and then cultured overnight. At 24, 48, 72, and 96 h, CRC cells with different transfections were supplemented with CCK-8 solution (10 μL/well) and cultured for an additional 2 h. Finally, the resulting optical density at 450 nm was measured using a microplate reader (Bio-Rad Laboratories, Hercules, CA, USA).

### Transwell assay

The transwell assays were conducted using transwell chambers (Corning Life Sciences, Corning, NY, USA) and matrigel (BD Biosciences, Franklin Lakes, NJ, USA). For the transwell migration assay, approximately 5 × 10^4^ transfected CRC cells suspended in 200 µL of medium without FBS were seeded in the upper chamber, while the lower chamber contained culture medium with 20% FBS as a chemoattractant. For cell invasion assay, cells were also seeded in the upper chamber precoated with matrigel. Next, the CRC cells with different transfections subjected to migration assay for 24 h and invasion assay for 48 h assay After removal of the cells remaining on the upper surface of the membrane, the cells migrated or invaded undersurface of the membrane were fixed. After staining with 0.1% crystal violet, the cells were observed and counted under a microscope.

### Colony formation assay

For the colony formation assay, 5 × 10^2^ transfected CRC cells were seeded into a six-well plate and cultured for 14 d. Afterward, the cell colonies were fixed with paraformaldehyde and stained with 0.1% crystal violet. Next, the plate was washed with PBS, and the total number of cell colonies of more than 50 cells was counted and photographed.

### Scratch wound healing assay

Transfected CRC cells were seeded in 6-well culture plates in complete DMEM/RPMI-1640 media and grown to 80% confluence in a monolayer overnight. Next, scratch wounds were inflicted along the diameter of each well using a 100-µL pipette tip. Thereafter, cell debris and suspended cells were removed by extensive washing, and then culture medium with 1% FBS was added. Finally, wound healing was measured and photographed under a microscope at 0 and 24 h within the wound line and the healing area was calculated using Image J software (NIH, Bethesda, MD, USA).

### 5-Ethynyl-2′-deoxyuridine (EdU) assay

A BeyoClick™ EdU-594 Cell Proliferation Kit (Beyotime Biotech) was used for the EdU assay. Briefly, the EdU reagent was directly dissolved into the culture medium at a 10-μM final concentration. After incubation for another 2 h, the CRC cells were fixed with paraformaldehyde for 15 min and stained with Click Additive Solution in the dark. Thereafter, Hoechst staining was used to counterstain nuclei, and EdU-positive cells were photographed and counted under a fluorescence microscope.

### Luciferase reporter assay

The targeted binding sites among circ_0003215, miR-663b, and DLG4 were confirmed by the dual-luciferase reporter assay. The binding sites between circ_0003215 and miR-663b, DLG4 and miR-663b were predicted by bioinformatics analysis. Luciferase vectors with circ_0003215 or the 3′-UTR of DLG4 and mutant types containing the firefly and Renilla luciferase genes were obtained from Promega (Madison, WI, USA). Next, HEK-293T cells were transfected with psiCHECK-circ_0003215 vectors (wild-type [WT] or mutant [MUT]) and miR-663b mimics, or psiCHECK-DLG4 vectors (WT1, WT2, or MUT1, MUT2) and miR-663b mimics. Relative luciferase activities were determined according to the corresponding instructions for the Dual-Luciferase Reporter Assay kit (Promega).

### RNA immunoprecipitation (RIP) assay

The RIP assay was conducted using the EZ-Magna RIP kit (MilliporeSigma, Burlington, MA, USA). Briefly, CRC cells were incubated with RIP lysis buffer containing RNase and protease inhibitors on ice for 60 min. Next, the cell lysates were incubated overnight with RIP buffer containing magnetic beads conjugated to IP-grade antibodies. Subsequently, the immune complexes were washed five times and treated with proteinase K. Finally, the immune precipitated RNA was isolated and subjected to RT-qPCR analysis.

### RNA pull-down assay

The biotinylated-circ_0003215 and NC probes were obtained from GenePharma (Shanghai, China). Briefly, CRC cells (1.5 × 10^7^) were fixed and lysed in lysis buffer. Afterward, the supernatant was collected, and 50 μL was pipetted for input after centrifugation, with the remainder incubated with biotin-labeled probes. Subsequently, the complexes were incubated with streptavidin-coupled Dynabeads overnight. Next, the beads that captured the immune complex were washed twice and then treated with proteinase K at 25 °C for 1 h. Finally, the bound RNAs were extracted using TRIzol, followed by RT-qPCR analysis. All probe sequences are listed in Supplementary Table S4.

### Oxidative PPP flux assay

Oxidative PPP flux was determined by measuring ^14^CO_2_ release. Briefly, a 10-cm dish with two sealed pinholes containing a 6-cm Petri dish was seeded with CRC cells. Afterward, the cells were cultured for 4 h with medium supplemented with [1-^14^C] or [6-^14^C]glucose until 80% confluence. The ^14^CO_2_ released from the cells was harvested by sealing the 10-cm dish. Oxidative PPP flux was stopped by injecting 2 N HCl through one of the holes, and the released ^14^CO_2_ was harvested for 12 h by injecting hyamine hydroxide into a cup placed on the 10-cm dish through the other hole. Subsequently, the hyamine hydroxide in the cup was dissolved in 60% methanol and subjected to scintillation counting. The ^14^CO_2_ content released was obtained by subtracting the scintillation count of the [6-^14^C] glucose treatment from that of the [1-^14^C]glucose treatment.

### ROS

The amount of intracellular ROS was quantified using dichlorodihydrofluorescein diacetate (DCFH‐DA; Invitrogen) oxidation. CRC cells transfected with specified plasmids were seeded into 6-well plates. After 24 h, the cells were washed and subsequently treated with 10 μM DCFH-DA for 30 min. Finally, the cells were harvested, resuspended in 500 µL FACS buffer, and subjected to FACS analysis (BD Biosciences).

### NADPH/NADP^+^ ratio

The NADPH/NADP^+^ ratio was determined using a Colorimetric Assay Kit (Sigma-Aldrich). Briefly, 3 × 10^6^ transfected cells were harvested and lysed with 200 μL NADP^+^/NADPH extraction buffer. Thereafter, the lysed cells were incubated for 5 min at 60 °C, and then 20 μL assay buffer and 200 μL counter NADPH/NADP^+^ extraction buffer were added. Next, the samples were centrifuged for 20 min, and the supernatants were used to determine the NADPH/NADP^+^ ratio. Finally, the absorbance at 565 nm was determined using a plate reader at 0 and 30 min.

### Protein half‑life assay

Cells were seeded in 6-well plates, and when they reached approximately 70% confluence, they were treated with the protein synthesis inhibitor cycloheximide at the indicated times. Afterward, the cells were harvested and analyzed by immunoblotting with specific antibodies.

### Immunoprecipitation

For immunoprecipitation, transfected cells were lysed using RIPA lysis buffer containing protease inhibitors on ice for 2 h. Next, 900 μL samples were incubated with protein A/G magnetic beads conjugated with immunoprecipitation antibodies at 4°C overnight. Finally, the bead-antibody complexes were washed thrice with RIPA lysis buffer, and the immuno-complexes were subjected to western blotting analysis.

### Ubiquitination assay

Cells were transfected with specific plasmids (HA-WT Ub, HA-K6O Ub, HA-K11O Ub, HA-K27O Ub, HA-K29O Ub, HA-K33O Ub, HA-K48O Ub, HA-K63O Ub, etc.). Forty-eight hours later, MG132 (10 μM; Sigma-Aldrich) was directly added to the culture media, and the cells were incubated for an additional 6h. Thereafter, the cells were collected, and the target protein were immunoprecipitated using A/G beads and IP-grade antibodies. Finally, the eluted proteins were analyzed by western blotting.

### Xenograft nude mouse model

Animal studies were approved by the Institutional Animal Care and Use Committee. All BALB/c mice were kept at the Wuhan Institute of Virology, Chinese Academy of Sciences (Wuhan, China), and were strictly and equally randomized. For the tumorigenesis assay, CRC cells with circ_0003215 overexpression or the respective control cells were suspended in 100 µL PBS and injected into the back of each mouse. Subcutaneous tumor volumes were estimated every 3d using the formula: volume = 1/2 × length × width^2^. Three weeks later, all mice were sacrificed, and subcutaneous tumors were excised and weighed. For the lung metastasis model, CRC cells (5 × 10^5^) with different transfections were injected via the caudal vein. All tail vein-injected mice were sacrificed after 6 weeks. Subsequently, their lungs were extracted and weighed, and the number of metastatic nodules was carefully calculated, experimenters were blinded from the treatment paradigm and where not involved in data analysis.

### Bioinformatics analysis

The sequence data of all circRNAs were searched and downloaded from circBank. The target miRNAs that directly interacted with circ_0003215 were predicted using circBank. Target miRNA genes were predicted using Targetscan, miRWalk, and miBase.

### Statistical analysis

All experiments were carried out independently at least thrice. The data are presented as mean ± standard deviation. Statistical analysis were conducted using SPSS 19.0 (SPSS Inc, Chicago, IL, USA) and GraphPad Prism v8.3.0 (GraphPad Software, San Diego, CA, USA). Before statistical analysis, the homogeneity of variance between groups was tested. The correlation between clinicopathologic parameters and circ_0003215 expression was analyzed using the chi-square test. Two-group comparisons of continuous variables were analyzed using the paired or unpaired Student’s t-test, all data followed a normal distribution. *P* < 0.05 was considered statistically significant.

## Supplementary information

Supplementary Tables

Supplementary Figures

Figure S1

Figure S2

Figure S3

Figure S4

Figure S5

Figure S6

Original Data File

aj-checklist

## Data Availability

The datasets analyzed during the current study were available from the corresponding authors.
