# Correction to: N6-methyladenosine modification of circ_0003215 suppresses the pentose phosphate pathway and malignancy of colorectal cancer through the miR-663b/DLG4/G6PD axis

**DOI:** 10.1038/s41419-022-05332-4

**Published:** 2022-10-18

**Authors:** Baoxiang Chen, Yuntian Hong, Rui Gui, Huabin Zheng, Shunhua Tian, Xiang Zhai, Xiaoyu Xie, Quanjiao Chen, Qun Qian, Xianghai Ren, Lifang Fan, Congqing Jiang

**Affiliations:** 1 Department of Colorectal and Anal Surgery, Zhongnan Hospital of Wuhan University, 430071 Wuhan, China; 2 Clinical Center of Intestinal and Colorectal Diseases of Hubei Province (Zhongnan Hospital of Wuhan University), 430071 Wuhan, China; 3 Hubei Key Laboratory of Intestinal and Colorectal Diseases (Zhongnan Hospital of Wuhan University), 430071 Wuhan, China; 4 Department of Infectious Diseases, Southwest Hospital, Third Military Medical University (Army Medical University), 400038 Chongqing, China; 5 CAS Key Laboratory of Special Pathogens and Biosafety, CAS Center for Influenza Research and Early Warning, Wuhan Institute of Virology, Chinese Academy of Sciences, 430064 Wuhan, China; 6 Department of Pathology, Zhongnan Hospital of Wuhan University, 430071 Wuhan, China

**Keywords:** Tumour biomarkers, Cancer metabolism, Colorectal cancer

Correction to: *Cell Death and Disease* 10.1038/s41419-022-05245-2, published online 20 September 2022

The original version of this article contained a mistake in the abstract. In the abstract, the sentence “The increased level of circ_ 0003215 was resulted from the RNA degradation by YTHDF2 is m6A reader protein” had to be changed to “The decreased level of circ_ 0003215 was resulted from the RNA degradation by the m6A reader protein YTHDF2”. The original article has been corrected.

