# Correction: N6-methyladenosine modification of circ_0003215 suppresses the pentose phosphate pathway and malignancy of colorectal cancer through the miR-663b/DLG4/G6PD axis

**DOI:** 10.1038/s41419-025-08199-3

**Published:** 2025-12-23

**Authors:** Baoxiang Chen, Yuntian Hong, Rui Gui, Huabin Zheng, Shunhua Tian, Xiang Zhai, Xiaoyu Xie, Quanjiao Chen, Qun Qian, Xianghai Ren, Lifang Fan, Congqing Jiang

**Affiliations:** 1 Department of Colorectal and Anal Surgery, Zhongnan Hospital of Wuhan University, 430071 Wuhan, China; 2 Clinical Center of Intestinal and Colorectal Diseases of Hubei Province (Zhongnan Hospital of Wuhan University), 430071 Wuhan, China; 3 Hubei Key Laboratory of Intestinal and Colorectal Diseases (Zhongnan Hospital of Wuhan University), 430071 Wuhan, China; 4 Department of Infectious Diseases, Southwest Hospital, Third Military Medical University (Army Medical University), 400038 Chongqing, China; 5 CAS Key Laboratory of Special Pathogens and Biosafety, CAS Center for Influenza Research and Early Warning, Wuhan Institute of Virology, Chinese Academy of Sciences, 430064 Wuhan, China; 6 Department of Pathology, Zhongnan Hospital of Wuhan University, 430071 Wuhan, China

Correction to: *Cell Death & Disease* 10.1038/s41419-022-05245-2, published online 20 September 2022

The original version of this article contained several errors. In the abstract, “Mechanismly” has been corrected to “Mechanistically.” In the figures, the following corrections were made: Figures 6E and S5E, “expresssion” corrected to “expression”; Figure S1C, “divergent primes” corrected to “divergent primers”; Figure S1E, “liner mRNA” corrected to “linear mRNA”; Figure S3C, “predicated miRNAs” corrected to “predicted miRNAs”; and several errors in Figure 5H, Figure 8E, and Supplementary Figure S6E have been corrected. We have carefully re-examined our original data and corrected these figures. We sincerely apologize for these errors and for any confusion they may have caused. The corrected figures are shown below, and these corrections do not affect the conclusions of the article. This study was approved by the Ethics Committee of Zhongnan Hospital of Wuhan University [No. 2020106]. The corrected original data have been updated in the PDF version of the Supplementary Original Files.

The original article has been corrected.

Figure 5 (Amended)
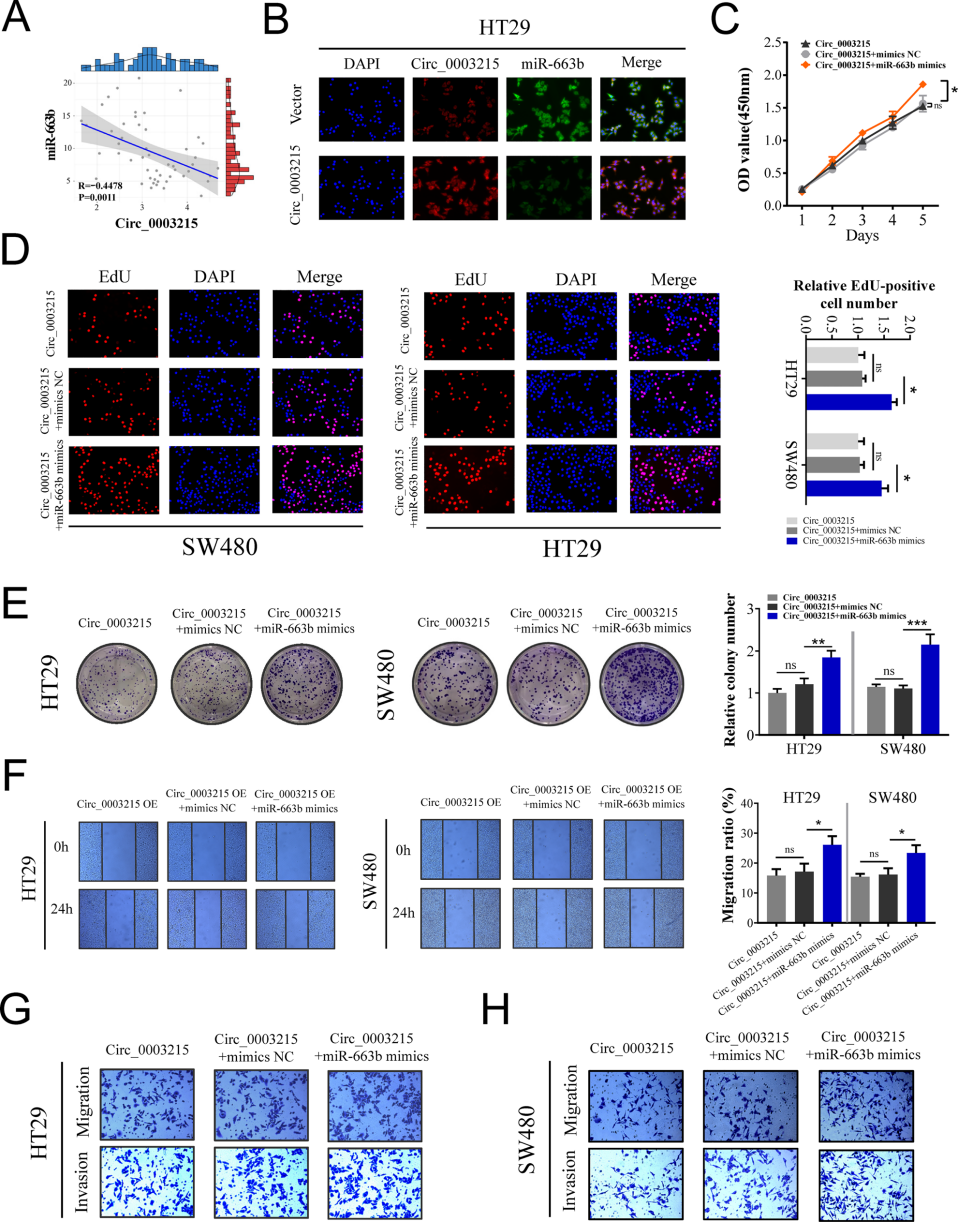


Figure 8 (Amended)
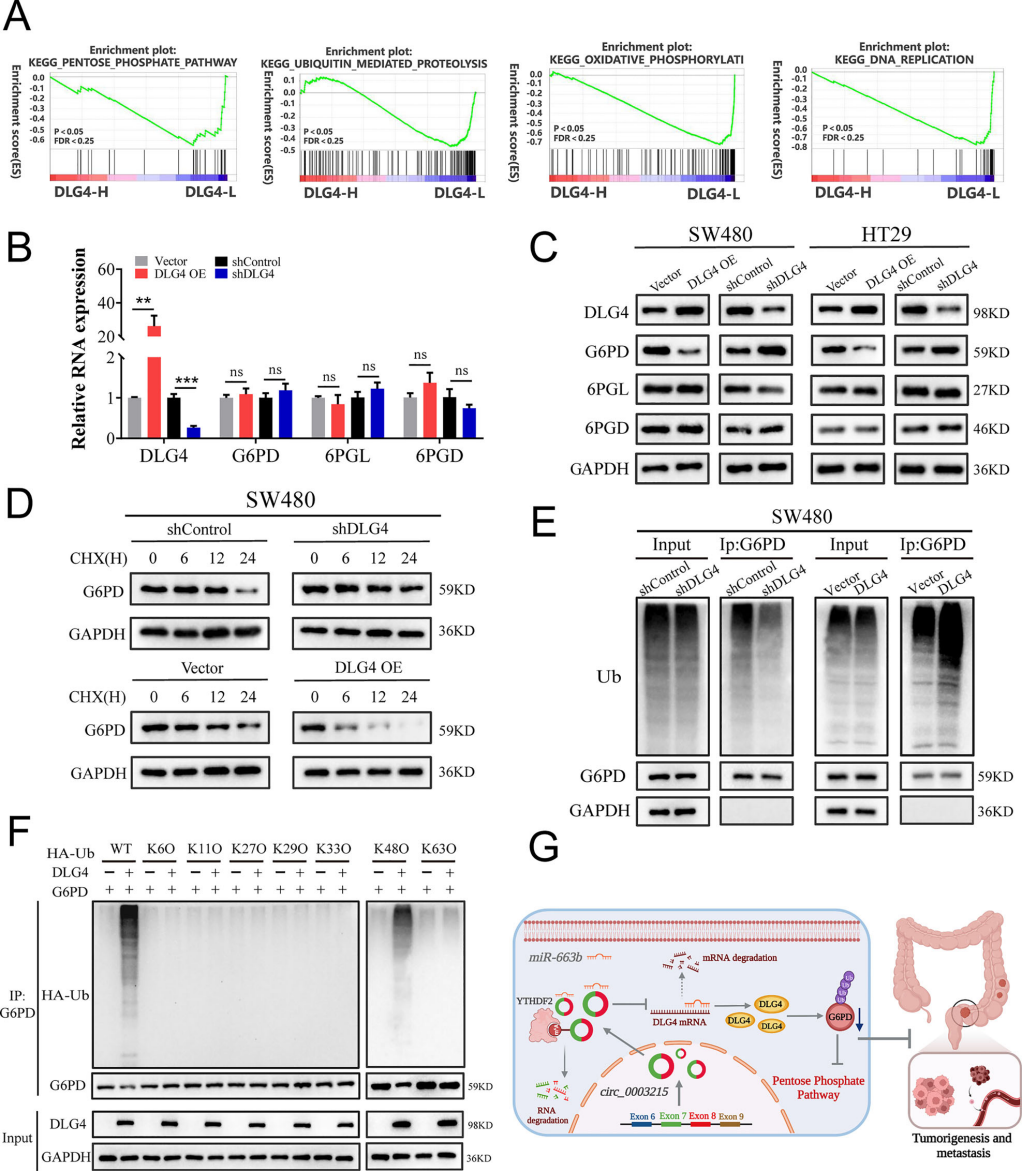


Figure S6 (Amended)
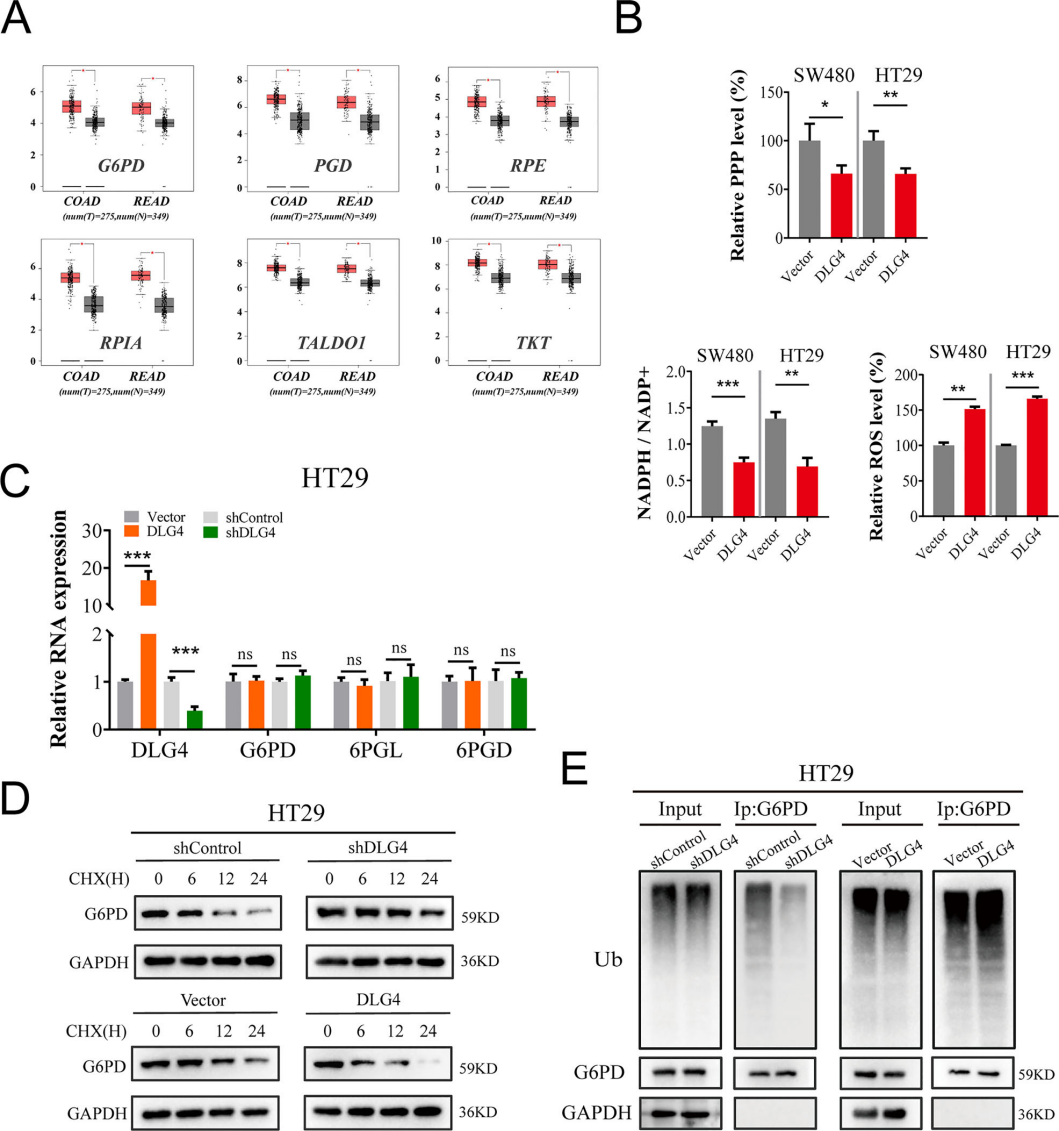


## Supplementary information

Supplementary Original files

