# Supplementary material for: N6-methyladenosine modification of circ_0003215 suppresses the pentose phosphate pathway and malignancy of colorectal cancer through the miR-663b/DLG4/G6PD axis
Source: Cell Death Dis. 2022 Sep 20;13(9):804. doi: 10.1038/s41419-022-05245-2 (PMC9489788; doi:10.1038/s41419-022-05245-2)
Supplement: Supplementary file 1 — Supplementary Tables [file 41419_2022_5245_MOESM1_ESM.docx]

**Table S1.** Characteristics of the patients’ cohort collected from100 patients

 diagnosed CRC.

| **No.** | **Age(y)** | **Gender** | **Tumor size**  **(diameter, cm)** | **Tumor site** | **Grade** | **TNM** | **Blood vessel invasion** |
| --- | --- | --- | --- | --- | --- | --- | --- |
| 1 | 61 | M | 6 | Colon | Low | T3N0M0 | Negative |
| 2 | 62 | F | 5 | Colon | Low | T3N1bM0 | Positive |
| 3 | 66 | F | 2.5 | Colon | Low | T3N2aM0 | Positive |
| 4 | 68 | M | 7 | Colon | Low | T4bN2bM0 | Positive |
| 5 | 66 | F | 2.9 | Colon | Low | T3N0M0 | Negative |
| 6 | 51 | M | 4 | Colon | Low | T3N0M0 | Negative |
| 7 | 64 | M | 5.5 | Colon | Low | T3N0M0 | Positive |
| 8 | 71 | F | 5 | Colon | High | T3N0M0 | Negative |
| 9 | 79 | M | 3.5 | Colon | Low | T3N0M0 | Negative |
| 10 | 77 | F | 4.5 | Rectum | Low | T3N1bM0 | Positive |
| 11 | 56 | M | 5 | Colon | Low | T3N0M0 | Negative |
| 12 | 58 | F | 5 | Colon | High | T4aN2aM0 | Positive |
| 13 | 68 | M | 8.5 | Colon | Low | T3N0M0 | Negative |
| 14 | 52 | F | 4 | Colon | Low | T2N1bM0 | Negative |
| 15 | 59 | F | 4 | Colon | Low | T3N0M0 | Positive |
| 16 | 79 | F | 3.5 | Colon | Low | T3N0M0 | Negative |
| 17 | 68 | M | 1.7 | Rectum | Low | T3N0M0 | Negative |
| 18 | 66 | M | 4 | Rectum | High | T3N2bM0 | Positive |
| 19 | 55 | F | 5 | Rectum | Low | T3N0M0 | Negative |
| 20 | 66 | M | 2 | Rectum | Low | T2N0M0 | Negative |
| 21 | 58 | M | 3 | Rectum | Low | T3N2aM0 | Positive |
| 22 | 57 | M | 5 | Rectum | Low | T3N2bM0 | Positive |
| 23 | 46 | M | 4 | Rectum | Low | T3N0M0 | Negative |
| 24 | 51 | M | 4 | Colon | High | T4N1aM0 | Positive |
| 25 | 40 | M | 4 | Colon | Low | T4N1cM0 | Positive |
| 26 | 87 | M | 4 | Rectum | Low | T3N2bM0 | Positive |
| 27 | 56 | F | 3 | Rectum | Low | T3N1bM0 | Positive |
| 28 | 60 | F | 8 | Colon | Low | T3N0M0 | Negative |
| 29 | 56 | F | 5 | Colon | High | T4aN1cM0 | Positive |
| 30 | 70 | M | 3 | Colon | High | T3N1M0 | Negative |
| 31 | 66 | M | 4 | Rectum | Low | T1N0M0 | Negative |
| 32 | 40 | M | 3.2 | Colon | Low | T3N0M0 | Negative |
| 33 | 53 | M | 3.5 | Rectum | High | T4N2M0 | Positive |
| 34 | 78 | F | 5 | Colon | Low | T2N0M0 | Negative |
| 35 | 66 | M | 7 | Colon | Low | T3N1M0 | Positive |
| 36 | 77 | M | 4.5 | Colon | High | T3N0M0 | Positive |
| 37 | 53 | F | 1.5 | Colon | Low | T2N0M0 | Negative |
| 38 | 84 | F | 4 | Rectum | Low | T3N2M0 | Positive |
| 39 | 65 | F | 5 | Rectum | Low | T3N1M0 | Positive |
| 40 | 21 | M | 3 | Rectum | Low | T2N0M0 | Negative |
| 41 | 58 | F | 7 | Colon | High | T3N1M0 | Negative |
| 42 | 58 | M | 4 | Rectum | Low | T2N0M0 | Negative |
| 43 | 52 | M | 5 | Rectum | High | T3N1M0 | Negative |
| 44 | 80 | M | 3 | Rectum | Low | T3N1M0 | Positive |
| 45 | 63 | F | 6 | Colon | Low | T4N1M0 | Positive |
| 46 | 54 | F | 3 | Rectum | Low | T3N0M0 | Negative |
| 47 | 48 | M | 6 | Colon | Low | T3N0M0 | Negative |
| 48 | 54 | F | 3 | Colon | Low | T3N2M0 | Negative |
| 49 | 71 | M | 3 | Rectum | Low | T1N1M0 | Negative |
| 50 | 60 | M | 6 | Rectum | Low | T2N0M0 | Negative |
| 51 | 71 | F | 4 | Colon | Low | T2N0M0 | Negative |
| 52 | 79 | M | 4 | Rectum | Low | T4N1M0 | Negative |
| 53 | 61 | M | 3 | Rectum | High | T2N2M0 | Positive |
| 54 | 64 | M | 3 | Colon | Low | T4N1M0 | Negative |
| 55 | 63 | F | 4 | Rectum | Low | T3N0M0 | Negative |
| 56 | 68 | F | 3.5 | Colon | Low | T3N1M0 | Positive |
| 57 | 56 | M | 4 | Rectum | Low | T3N1cM1 | Positive |
| 58 | 45 | M | 3.5 | Rectum | High | T3N2bM1 | Positive |
| 59 | 80 | F | 2.5 | Rectum | Low | T2N0M0 | Negative |
| 60 | 62 | F | 4.5 | Colon | Low | T3N1aM1 | Negative |
| 61 | 52 | F | 6 | Colon | Low | T3N0M0 | Negative |
| 62 | 64 | M | 3.5 | Colon | Low | T3N1aM0 | Positive |
| 63 | 78 | M | 8 | Colon | Low | T3N0M0 | Negative |
| 64 | 57 | F | 6 | Rectum | Low | T3N0M0 | Negative |
| 65 | 67 | M | 4 | Rectum | Low | T3bN1aM0 | Negative |
| 66 | 88 | M | 2.5 | Colon | Low | T3N0M0 | Negative |
| 67 | 47 | F | 3.5 | Rectum | Low | T3N1cM0 | Positive |
| 68 | 72 | M | 3 | Colon | Low | T3N1aM1 | Positive |
| 69 | 46 | M | 4 | Rectum | Low | T4aN2aM1c | Positive |
| 70 | 59 | M | 2.2 | Rectum | High | T2N0M0 | Negative |
| 71 | 61 | M | 4 | Rectum | Low | T2N0M0 | Negative |
| 72 | 70 | M | 1.5 | Rectum | Low | T2N0M0 | Negative |
| 73 | 62 | F | 6 | Colon | Low | T3N2bM1 | Positive |
| 74 | 70 | M | 5.5 | Rectum | High | T3N2M0 | Positive |
| 75 | 83 | F | 2 | Colon | Low | T2N0M0 | Negative |
| 76 | 49 | F | 3.5 | Colon | Low | T4bN1M1 | Negative |
| 77 | 75 | M | 1.6 | Rectum | Low | T2N0M0 | Positive |
| 78 | 63 | M | 3.5 | Colon | Low | T4N1M0 | Negative |
| 79 | 48 | M | 4.5 | Colon | Low | T3N0M0 | Negative |
| 80 | 58 | M | 5 | Rectum | Low | T3N1aM0 | Negative |
| 81 | 55 | F | 4.5 | Colon | Low | T4bN0M1 | Negative |
| 82 | 27 | M | 4.5 | Colon | High | T4aN1aM0 | Positive |
| 83 | 62 | M | 4 | Rectum | Low | T3N1cM0 | Negative |
| 84 | 59 | M | 4 | Rectum | Low | T3N1bM0 | Negative |
| 85 | 42 | M | 3.5 | Colon | Low | T4aN1M0 | Positive |
| 86 | 56 | F | 2.2 | Rectum | Low | T3N1M0 | Positive |
| 87 | 68 | M | 2.5 | Rectum | Low | T3N0M0 | Negative |
| 88 | 44 | F | 3.5 | Colon | Low | T3N1M0 | Positive |
| 89 | 46 | F | 8 | Colon | Low | T3N0M0 | Negative |
| 90 | 84 | M | 6.5 | Rectum | Low | T3N1cM0 | Negative |
| 91 | 79 | F | 2.5 | Colon | Low | T3N1M0 | Negative |
| 92 | 64 | M | 3.8 | Rectum | Low | T3N0M0 | Negative |
| 93 | 68 | M | 3.5 | Colon | Low | T3N0M1 | Negative |
| 94 | 83 | F | 7.5 | Colon | Low | T4bN0M0 | Negative |
| 95 | 26 | M | 6.5 | Colon | High | T3N0M0 | Negative |
| 96 | 57 | M | 2.2 | Colon | Low | T3N1bM0 | Negative |
| 97 | 62 | M | 3.5 | Rectum | Low | T3N0M0 | Negative |
| 98 | 68 | M | 5.5 | Rectum | Low | T3N1cM0 | Negative |
| 99 | 48 | F | 4.5 | Colon | Low | T2N0M0 | Negative |
| 100 | 62 | M | 5 | Colon | Low | T3N2M0 | Negative |

**Table S2.** The primers used in this study.

| hsa_circ_0003215 | Forward | AGCGAGAAATCTTGGTGGAGG |
| --- | --- | --- |
|  | Reverse | AGGACGGCAAAAATCCTCGC |
| hsa_circ_0001955 | Forward | TCGAAATCAGGTGAAGGTCTCC |
|  | Reverse | CAAGTCCTCCAAGCTAGGGC |
| hsa_circ_0024824 | Forward | AAGATTAAGCAGGTGGCCCG |
|  | Reverse | AACATGGCCAGGAGCTTCC |
| hsa_circ_0041481 | Forward | TGCTGTTTTCTTGGGGGACA |
|  | Reverse | GGACACAGATTCACCCGTCA |
| circ_NSUN2 | Forward | CCACCACTGCTCCTCAAC |
|  | Reverse | AAGTGTGGCGATTTTCTCAAG |
| Convergent-circ_0003215 | Forward | AAGAGAGCTACAGGCCGAGA |
|  | Reverse | AAGCTTGTCGTTGACGGTCA |
| MYO9B | Forward | GGCCTCGGAAGGTCAGTATTA |
|  | Reverse | GACCGTTACGAAGGACGACG |
| GAPDH | Forward | CCAGCAAGAGCACAAGAGGA |
|  | Reverse | ACATGGCAACTGTGAGGAGG |
| Divergent GAPDH | Forward | GAAGGTGAAGGTCGAGTC |
|  | Reverse | GAAGATGGTGATGGGATTTC |
| U6 | Forward | CTCGCTTCGGCAGCACA |
|  | Reverse | AACGCTTCACGAATTTGCGT |
| DLG4 | Forward | AGTGACAACCAAGAAATACCGCT |
|  | Reverse | CCCTCTGTTCCATTCACCTGC |
| STXBP5L | Forward | GCTGGAAGTGGTTCCGTACAT |
|  | Reverse | GAACTGGATCAAAGGCTAATGCT |
| SYT2 | Forward | CAGGCTAATCAGCTTACTGTGG |
|  | Reverse | TGTCTTCCGATGGACTTTGGT |
| DCX | Forward | TCCCGGATGAATGGGTTGC |
|  | Reverse | GCGTACACAATCCCCTTGAAGTA |
| SHANK2 | Forward | CTTTGGATTCGTGCTTCGAGG |
|  | Reverse | GACTCCAGGTACTGTAGGGC |
| IQSEC2 | Forward | GAGAAGGAAGCGGGCTATTCG |
|  | Reverse | TCCACACTGACTGTTCTGGAA |
| MAPT | Forward | TCCACACTGACTGTTCTGGAA |
|  | Reverse | CCAATCTTCGACTGGACTCTGT |
| G6PD | Forward | ACGACGAAGCGCAGACAG |
|  | Reverse | TCCGACTGATGGAAGGCATC |
| 6PGL | Forward | TGCAGCACGAACTGTCATCT |
|  | Reverse | CTGGCCAGCTACAAAGTGGA |
| 6PGD | Forward | GTGCCCAGTCCCTGAAAGAG |
|  | Reverse | TCTCGGCACCGTCTCAATTT |
| hsa-miR-2392‐RT | GTCGTATCCAGTGCAGGGTCCGAGGTATTCGCACTGGATACGACCACCTC | |
| hsa-miR-3615‐RT | GTCGTATCCAGTGCAGGGTCCGAGGTATTCGCACTGGATACGACGAGCCG | |
| hsa-miR-3655‐RT | GTCGTATCCAGTGCAGGGTCCGAGGTATTCGCACTGGATACGACAGCAAC | |
| hsa-miR-3667-3p‐RT | GTCGTATCCAGTGCAGGGTCCGAGGTATTCGCACTGGATACGACAAAGAC | |
| hsa-miR-4533‐RT | GTCGTATCCAGTGCAGGGTCCGAGGTATTCGCACTGGATACGACAGCGTC | |
| hsa-miR-4726-3p‐RT | GTCGTATCCAGTGCAGGGTCCGAGGTATTCGCACTGGATACGACTGCGGC | |
| hsa-miR-4747-5p‐RT | GTCGTATCCAGTGCAGGGTCCGAGGTATTCGCACTGGATACGACCTAAGA | |
| hsa-miR-4790-5p‐RT | GTCGTATCCAGTGCAGGGTCCGAGGTATTCGCACTGGATACGACAACATG | |
| hsa-miR-5196-5p‐RT | GTCGTATCCAGTGCAGGGTCCGAGGTATTCGCACTGGATACGACCCCAAC | |
| hsa-miR-663b-RT | GTCGTATCCAGTGCAGGGTCCGAGGTATTCGCACTGGATACGACGCCTCA | |
| hsa-miR-6735-3p‐RT | GTCGTATCCAGTGCAGGGTCCGAGGTATTCGCACTGGATACGACCTGAGG | |
| hsa-miR-6801-5p‐RT | GTCGTATCCAGTGCAGGGTCCGAGGTATTCGCACTGGATACGACTCATTT | |
| hsa-miR-6884-5p‐RT | GTCGTATCCAGTGCAGGGTCCGAGGTATTCGCACTGGATACGACCAACAT | |
| U6-RT | GTCGTATCCAGTGCAGGGTCCGAGGTATTCGCACTGGATACGACAAAATA | |
| hsa-miR-2392 | Forward | CGCGTAGGATGGGGGTGA |
| hsa-miR-3615 | Forward | GCGTCTCTCGGCTCCTCG |
| hsa-miR-3655 | Forward | CGGCTTGTCGCTGCGGT |
| hsa-miR-3667-3p | Forward | GCGACCTTCCTCTCCATGG |
| hsa-miR-4533 | Forward | CGTGGAAGGAGGTTGCCG |
| hsa-miR-4726-3p | Forward | CGACCCAGGTTCCCTCTG |
| hsa-miR-4747-5p | Forward | CGAGGGAAGGAGGCTTGG |
| hsa-miR-4790-5p | Forward | GCGCGATCGCTTTACCATT |
| hsa-miR-5196-5p | Forward | CGAGGGAAGGGGACGAGG |
| hsa-miR-663b | Forward | GGTGGCCCGGCCGTGC |
| hsa-miR-6735-3p | Forward | CGAGGCCTGTGGCTCCTC |
| hsa-miR-6801-5p | Forward | CGTGGTCAGAGGCAGCAGG |
| hsa-miR-6884-5p | Forward | CGCGAGAGGCTGAGAAGGTG |
| U6 | Forward | ACAGAGAAGATTAGCATGGCCC |
| Universal 5’ primer | Reverse | AGTGCAGGGTCCGAGGTATT |

**Table S3.** The primary antibodies used in this study.

| Antibody | Supplier | Catalogue | Host |
| --- | --- | --- | --- |
| AGO2 | Abcam | ab186733 | Rabbit |
| YTHDF1 | Proteintech | 17479-1-AP | Rabbit |
| YTHDF2 | Proteintech | 24744-1-AP | Rabbit |
| YTHDF3 | Proteintech | 25537-1-AP | Rabbit |
| YTHDC1 | Proteintech | 29441-1-AP | Rabbit |
| m6A | Proteintech | 68055-1-Ig | Mouse |
| GAPDH | Proteintech | 60004-1-Ig | Mouse |
| IgG | Abcam | ab172730 | Rabbit |
| DLG4 | Proteintech | Ab238135 | Rabbit |
| G6PD | [ABclonal](https://www.baidu.com/link?url=BT_CNMcdaLvzAqDN1HhfwXsJil-oG4j2Jy3nWptRe0RTj7S51195P_dAgMdOGtjc&wd=&eqid=bf2dae4a0016ba210000000661fd02b3) | A0563 | Rabbit |
| 6PGL | [ABclonal](https://www.baidu.com/link?url=BT_CNMcdaLvzAqDN1HhfwXsJil-oG4j2Jy3nWptRe0RTj7S51195P_dAgMdOGtjc&wd=&eqid=bf2dae4a0016ba210000000661fd02b3) | A8366 | Rabbit |
| 6PGD | [ABclonal](https://www.baidu.com/link?url=BT_CNMcdaLvzAqDN1HhfwXsJil-oG4j2Jy3nWptRe0RTj7S51195P_dAgMdOGtjc&wd=&eqid=bf2dae4a0016ba210000000661fd02b3) | A7710 | Rabbit |
| Ubiquitin | Proteintech | 10201-2-AP | Rabbit |
| HA | Proteintech | 51064-2-AP | Rabbit |

**Table S4.** siRNA and RNA oligonucleotides sequences.

| Name | Sequences |
| --- | --- |
| Si1-circ_0003215 | [UACAGCCUCAGCGAGGAUUUU](http://blast.ncbi.nlm.nih.gov/Blast.cgi?PROGRAM=blastn&PAGE_TYPE=BlastSearch&LINK_LOC=blasthome&QUERY=>hsa_circ_0003215-siRNA2%0ATACAGCCTCAGCGAGGATTTT&DATABASE=nr&EQ_MENU=Homo%C2%A0sapiens%C2%A0(taxid:9606)) |
| Si2-circ_0003215 | AGCCUCAGCGAGGAUUUUU |
| Si3-circ_0003215 | [GCGAGGAUUUUUGCCGUCCUC](http://blast.ncbi.nlm.nih.gov/Blast.cgi?PROGRAM=blastn&PAGE_TYPE=BlastSearch&LINK_LOC=blasthome&QUERY=>hsa_circ_0003215-siRNA9%0AGCGAGGATTTTTGCCGTCCTC&DATABASE=nr&EQ_MENU=Homo%C2%A0sapiens%C2%A0(taxid:9606)) |
| miR-663b mimics | GGUGGCCCGGCCGUGCCUGAGG |
| miR-663b inhibitor | CCUCAGGCACGGCCGGGCCACC |
| miR-663b-FAM  for FISH | UCAGGCACGGCCGGGCCA |
| Circ_0003215 probe for RNA pull-down | AGAGGA+CGGCAAAAA+UCCUCGCUGAGGC+UGUAGG |
| NC probe for RNA pull-down | UUGUACUACACAAAAGUACUG |
