# Supplementary material for: N6-methyladenosine modification of circ_0003215 suppresses the pentose phosphate pathway and malignancy of colorectal cancer through the miR-663b/DLG4/G6PD axis
Source: Cell Death Dis. 2022 Sep 20;13(9):804. doi: 10.1038/s41419-022-05245-2 (PMC9489788; doi:10.1038/s41419-022-05245-2)
Supplement: Supplementary file 2 — Supplementary Figures [file 41419_2022_5245_MOESM2_ESM.docx]

**
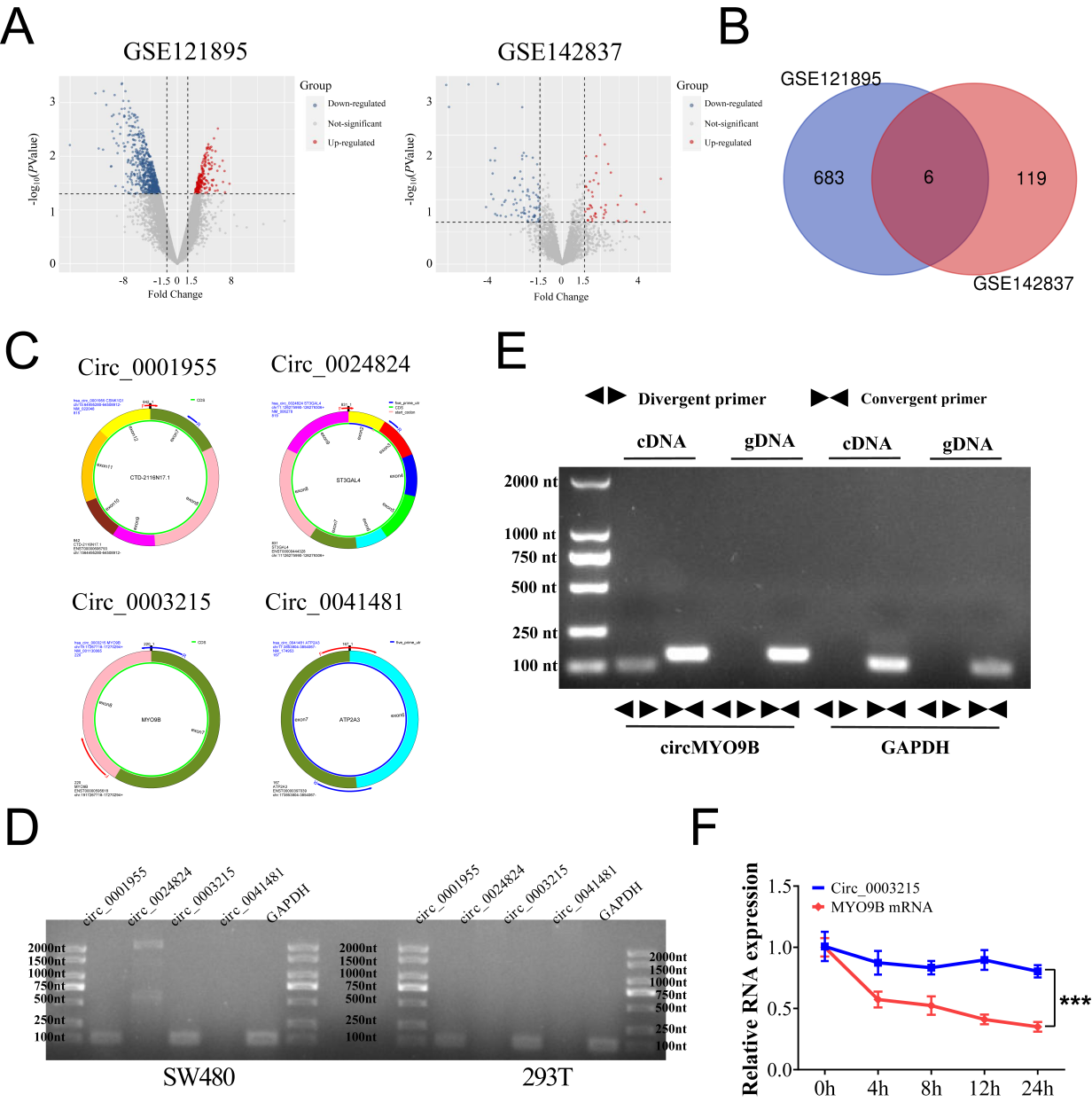
**

**Figure S1.** Expression profiles of circRNAs. **(A)** Volcano plots showing the upregulated(red) and downregulated(blue) circRNAs in CRC from GSE121895 and GSE142837. **(B)** Venn diagram exhibiting the overlap of differentially expressed circRNAs in GSE121895 and GSE142837. **(C)** The divergent primes of circRNAs were verified by circPrimer. **(D)** RT-PCR assay with divergent primers showing the differentially expressed circRNAs in CRC cell line (SW480, left panel) and HEK-293T (right panel). **(E)** The product of circ_0003215 and liner mRNA amplified by the convergent or divergent primers in HT29 cells by agarose gel electrophoresis. **(F)** Time-course qRT-PCR analysis of circ_0003215 and its linear counterpart MYO9B in HT29 cells after actinomycin D treatment.


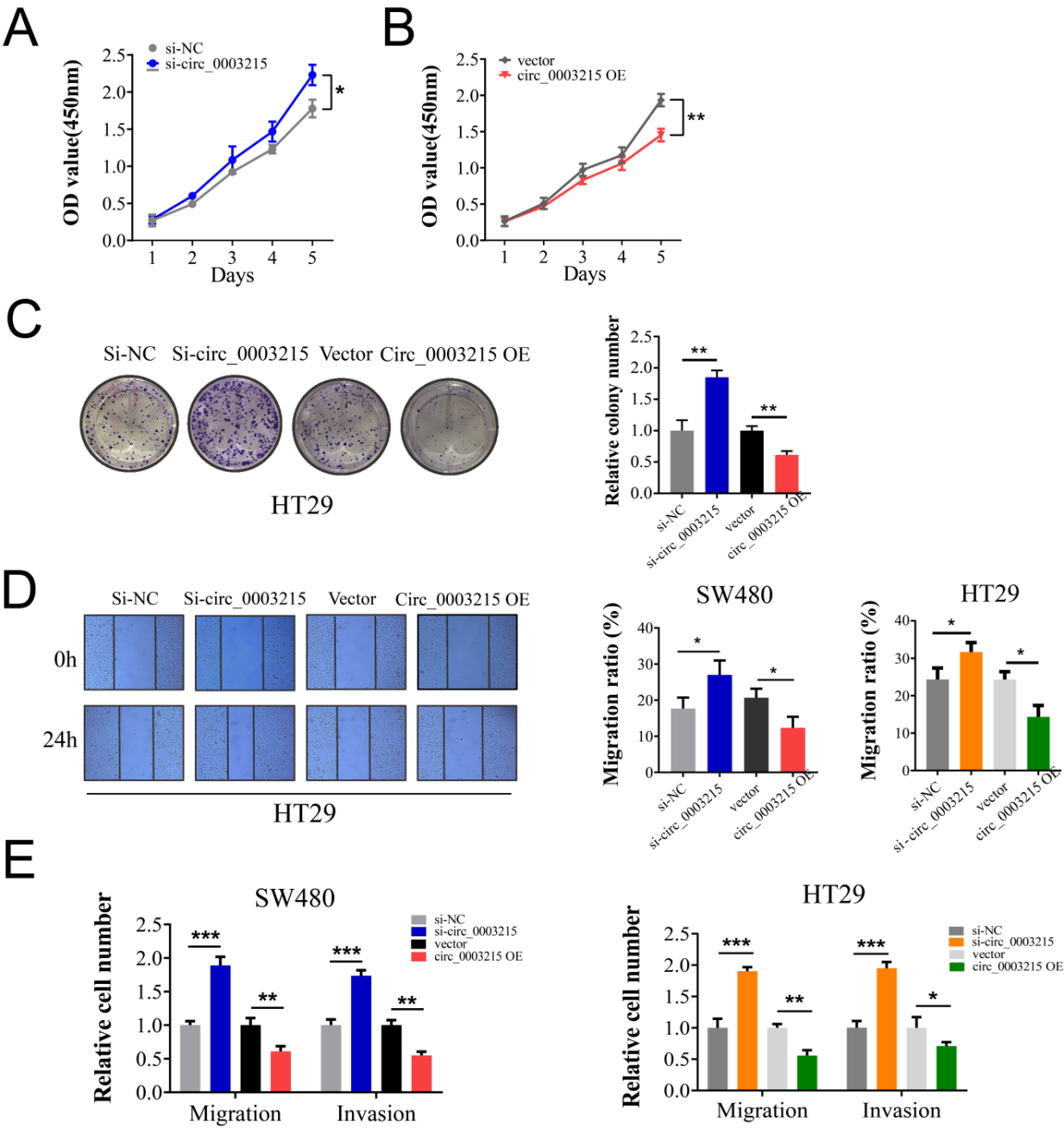


**Figure S2.** Circ_0003215 suppresses CRC cell proliferation, invasion and migration in vitro. **(A,B)** CCK-8 assay was performed to evaluate cell proliferative effects of circ_0003215 in SW480. **(C)** Colony formation assay of HT29 cells transfected with control, circ_0003215 siRNA, vector or pLCDH-circ_0003215. **(D)** Wound healing assay to detect the effect of circ_0003215 on cell migration of HT29 and SW480. **(E)** Statistical analysis of transwell assay to detect the effect of circ_0003215 on cell migration of HT29. Graph represents mean±SD; **P* < 0.05, ***P* < 0.01, ****P* < 0.001.

**
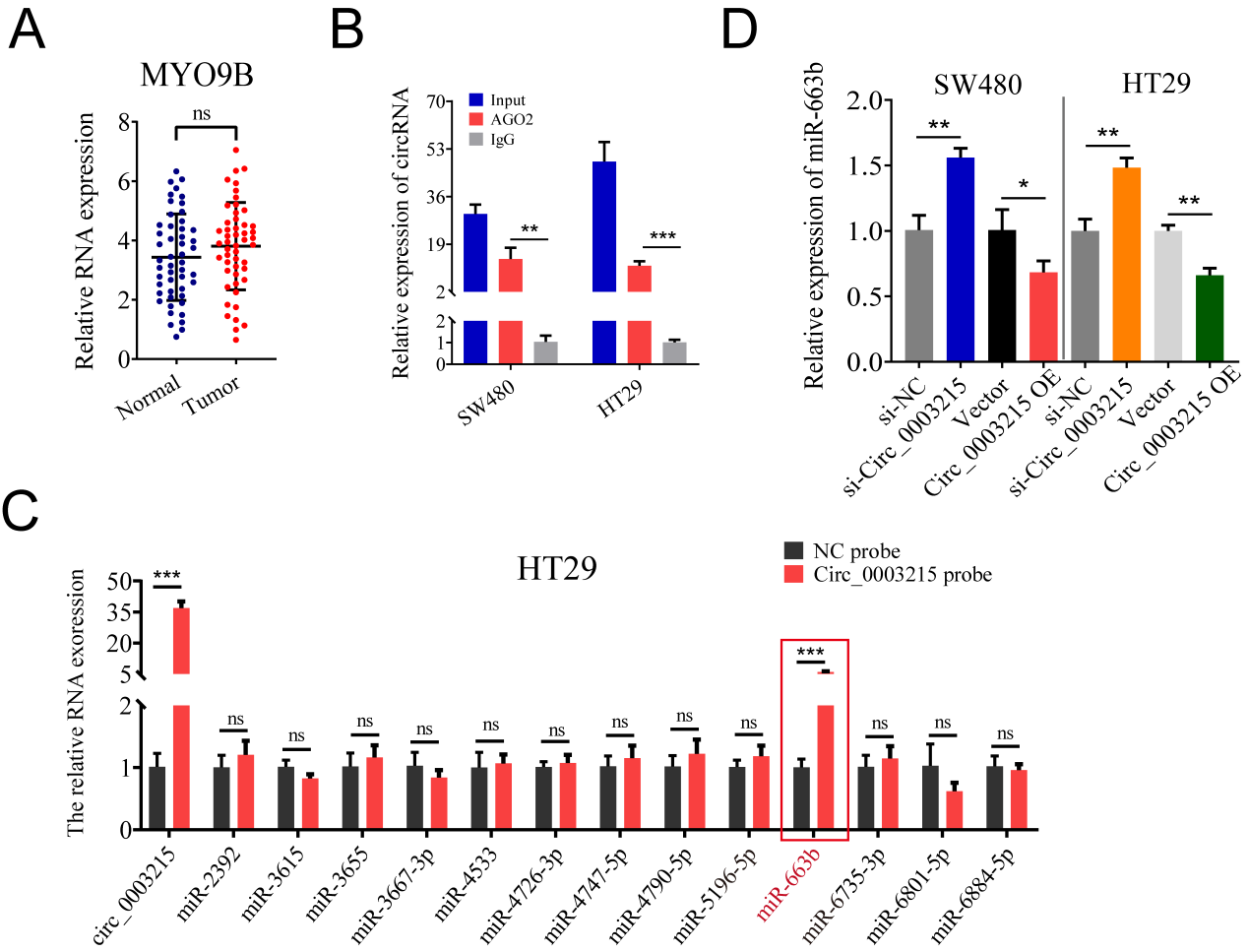
**

**Figure S3.** Circ_0003215 functions as an efficient miR-663b sponge in CRC cells. **(A)** The expression of linear MYO9B mRNA in 50 pairs of CRC tissues. **(B)** The expression of circ_0003215 was analysed by RT-qPCR in CRC cell lysates enriched with anti-AGO2 antibody immunoprecipitate. **(C)** The relative RNA expression of circ_0003215 and predicated miRNAs were detected by qRT-PCR in HT29 cells after RNA pull-down. Results were presented as the percentage of pull-down to input. **(D)** The expression of miR-663b in SW480 and HT29 cells transfected with circ_0003215-specific siRNA or overexpression plasmids. Graph are plotted as the mean± SD. **P* < 0.05, ***P* < 0.01, ****P* < 0.001.

**
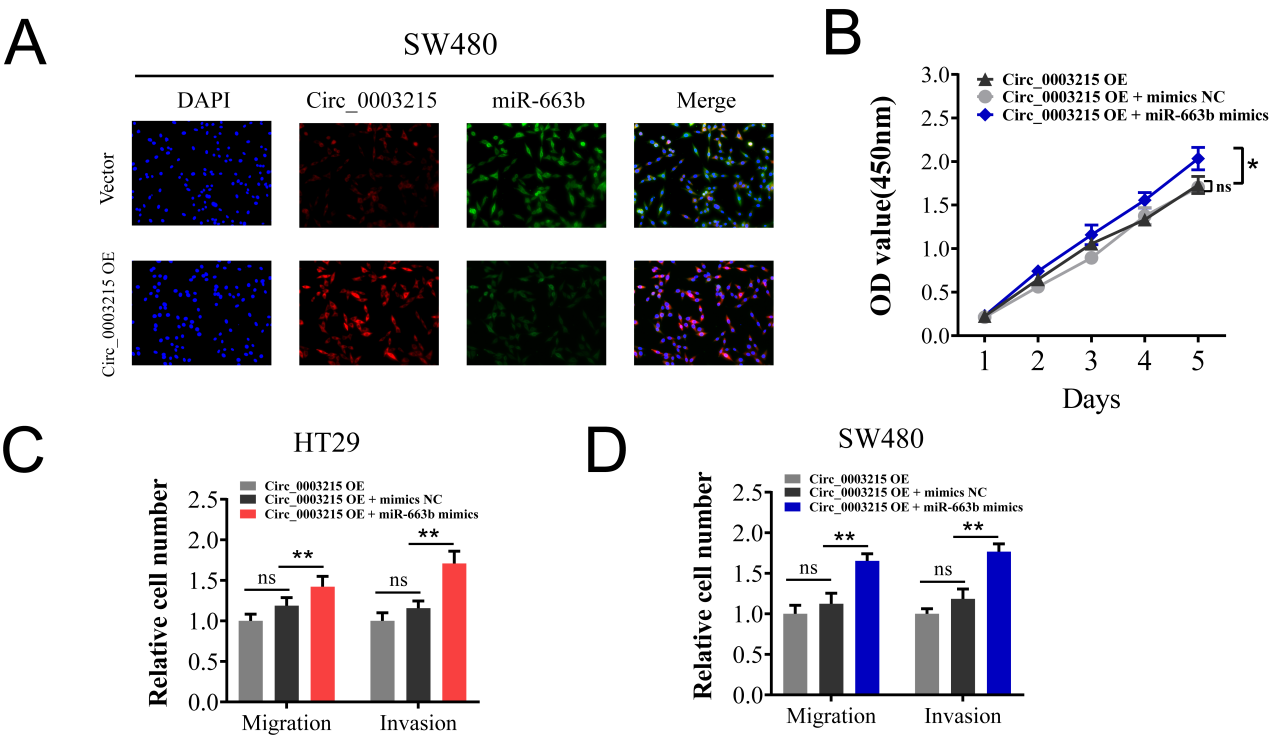
**

**Figure S4.** miR-663b can reverse the effects of circ_0003215 on the biofunction in CRC cells. **(A)** FISH analysis of circ_0003215 (red) and miR-663b (green) in SW480 cells transfected with vector or circ_0003215 plasmid. **(B)** CCK8 analysis of the cell proliferation ability in SW480 cells transfected with indicated vector. **(C,D)** Statistical analysis of wound healing assay to detect the effect on cell migration of SW480 cells transfected with indicated vector. Graph represents mean±SD; **P* < 0.05, ***P* < 0.01, ****P* < 0.001.


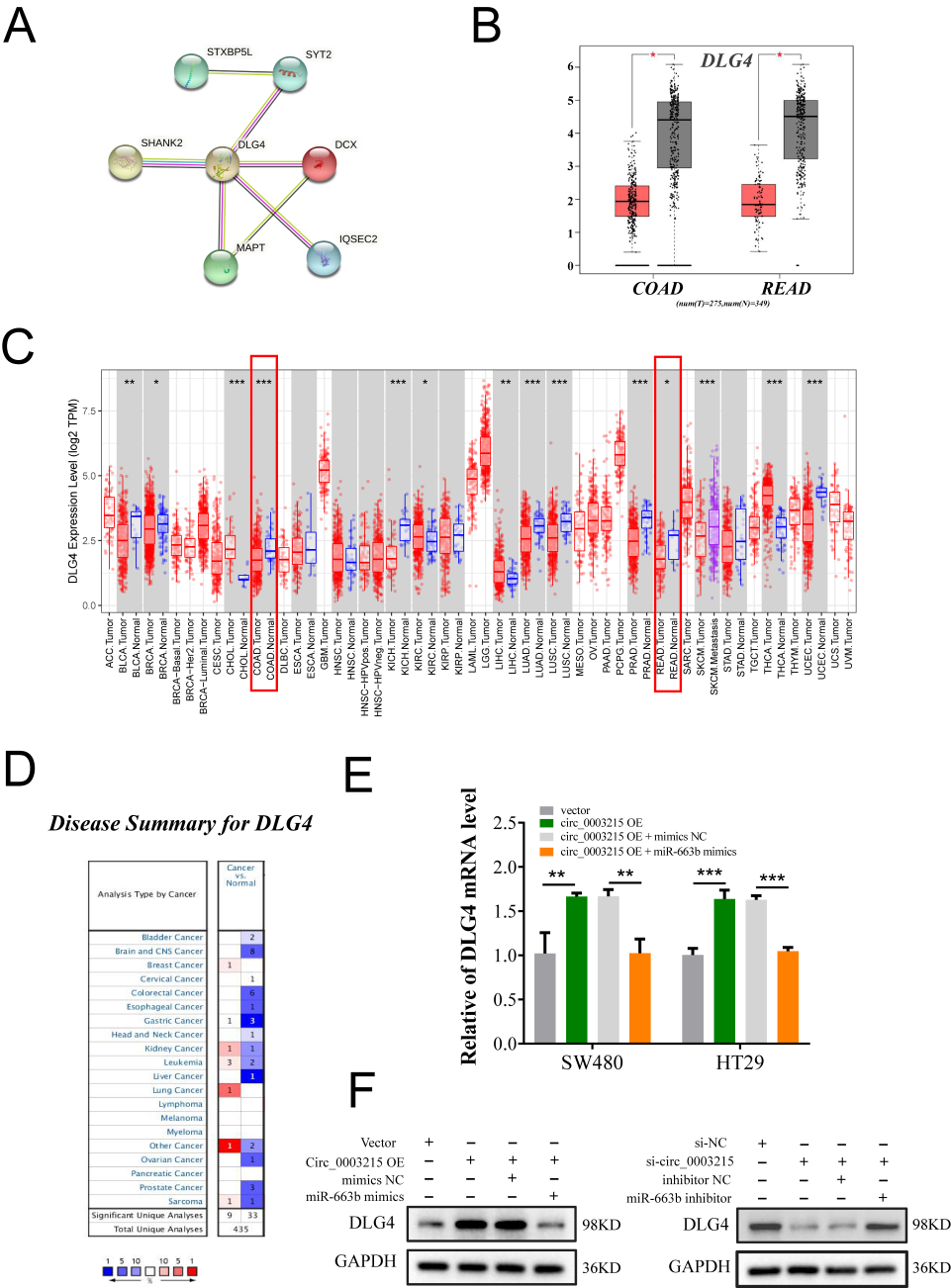


**Figure S5.** DLG4 is a direct target of circ_0003215/miR-663b axis in CRC cells. **(A)** The coexpression analysis of the 7 predicted hub genes using the STRING online database. **(B)** The expression of DLG4 in colon cancer (COAD) and rectal cancer (READ) dataset was analyzed by gene expression profiling interactive analysis (GEPIA). **(C)** Evaluation of DLG4 expression level in different types of tumor tissues and normal tissues in TIMER database. **(D)** DLG4 expression levels in multiple cancers from Oncomine Database. **(E)** The expresssion level of DLG4 mRNA in SW480 and HT29 cells transfected with circ_0003215 or miR-663b mimics in SW480 and HT29 cells. **(F)** Western blotting of DLG4 was detected in HT29 cells transfected with indicated vectors.

**
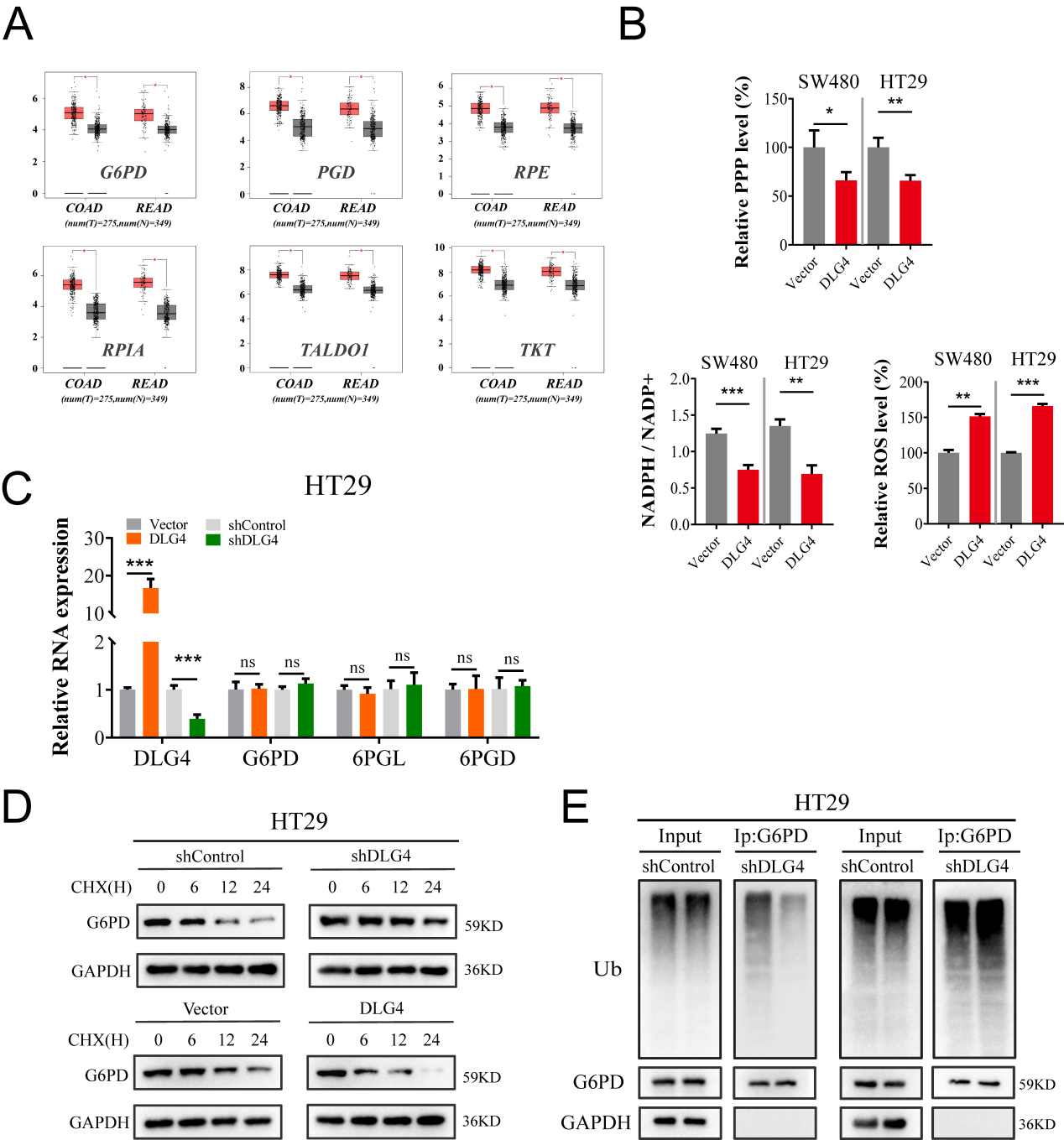
**

**Figure S6.** DLG4 regulates PPP through the K48-linked ubiquitination of G6PD. **A** Six key enzymes of the PPP were highly expressed in CRC samples according to the GEPIA database. **B** The NADPH/NADP+ ratio, oxidative PPP flux, and intracellular ROS levels were measured in CRC cells with DLG4 overexpression. **C** The mRNA levels of DLG4, G6PD, 6PGL and 6PGD were detected in HT29 cells using RT-qPCR, upon overexpression or knockdown of DLG4. **D** Degradation of G6PD was investigated using western blot analysis in a CHX chase assay in SW480 cells after DLG4 overexpression or knockdown. **E** The immunoprecipitation/western blotting assays were used to detect the ubiquitination levels of G6PD protein in HT29 cells after overexpression or knockdown of DLG4.
