# Supplementary figures and images for: N6-methyladenosine modification of circ_0003215 suppresses the pentose phosphate pathway and malignancy of colorectal cancer through the miR-663b/DLG4/G6PD axis
Source: Cell Death Dis. 2022 Sep 20;13(9):804. doi: 10.1038/s41419-022-05245-2 (PMC9489788; doi:10.1038/s41419-022-05245-2)

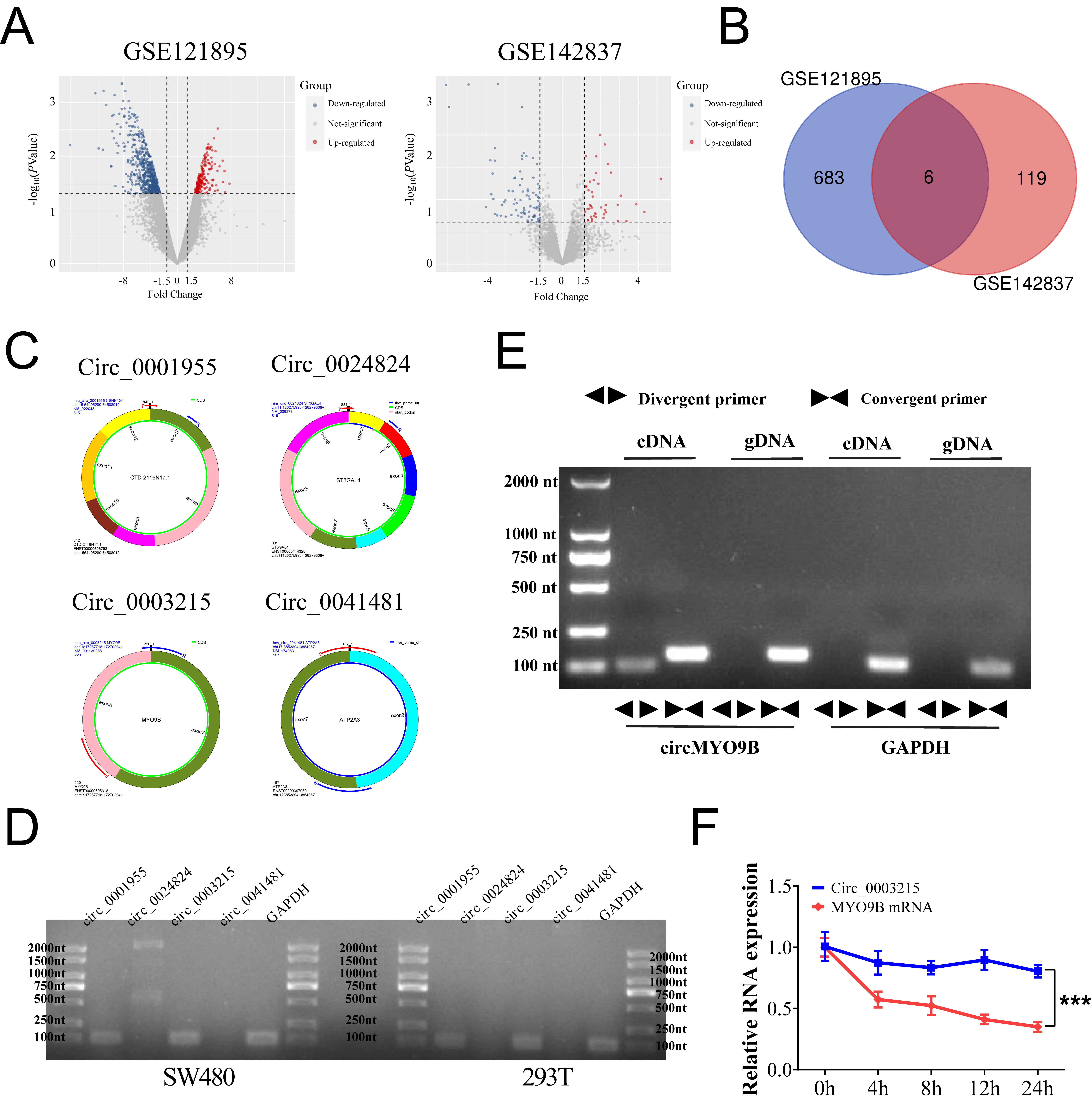

Supplement: Supplementary file 3 — Figure S1 [file 41419_2022_5245_MOESM3_ESM.png]

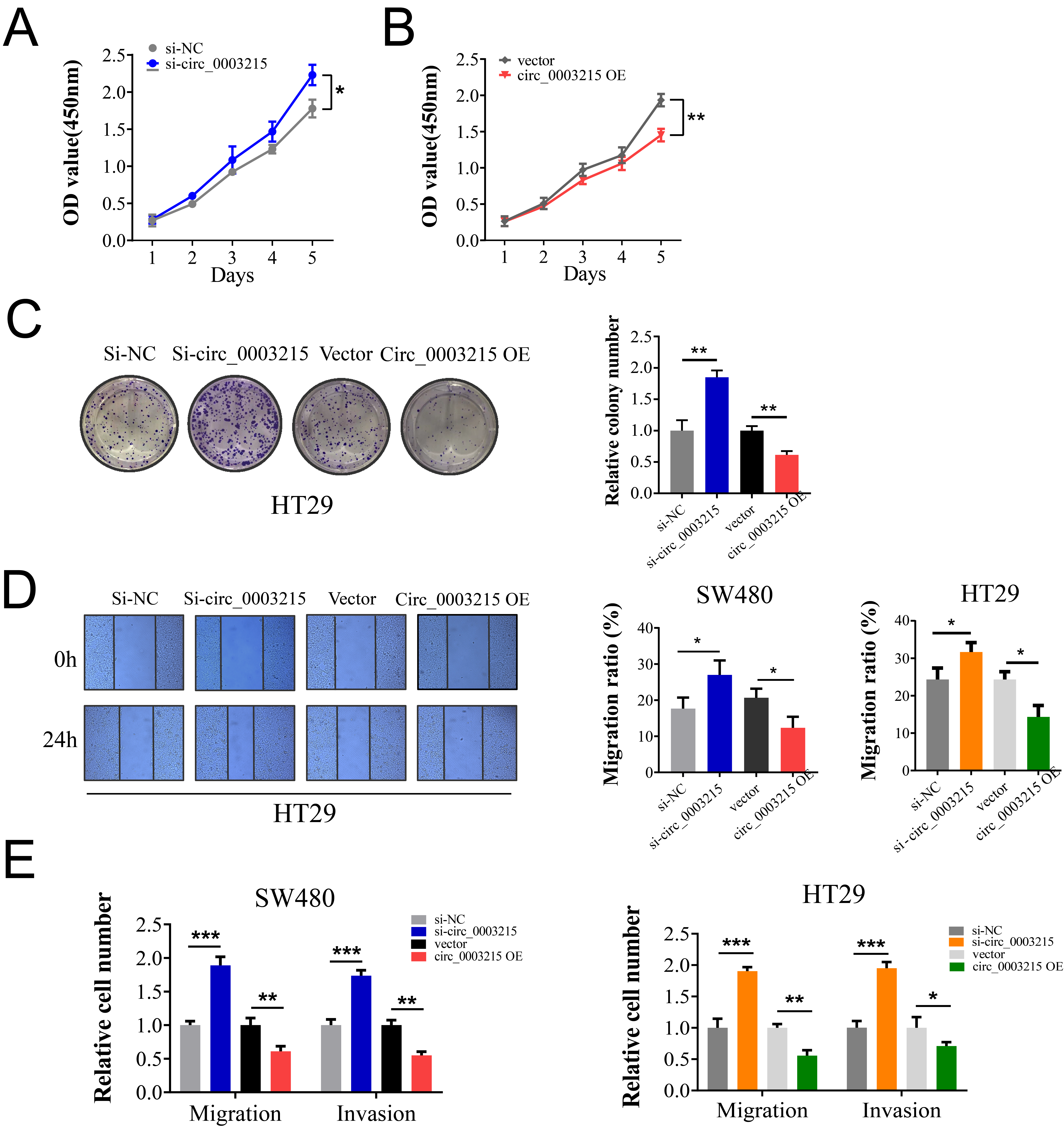

Supplement: Supplementary file 4 — Figure S2 [file 41419_2022_5245_MOESM4_ESM.png]

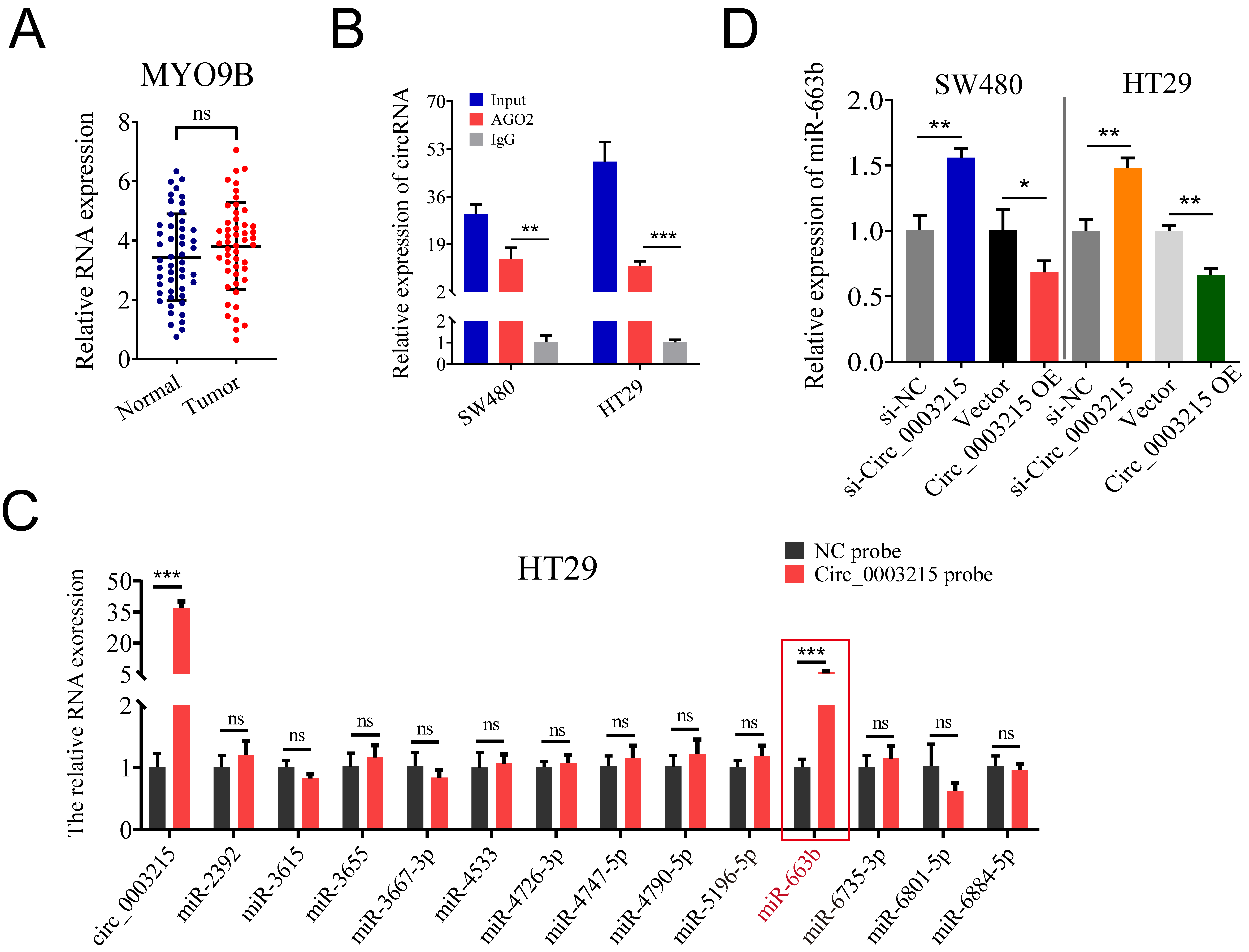

Supplement: Supplementary file 5 — Figure S3 [file 41419_2022_5245_MOESM5_ESM.png]

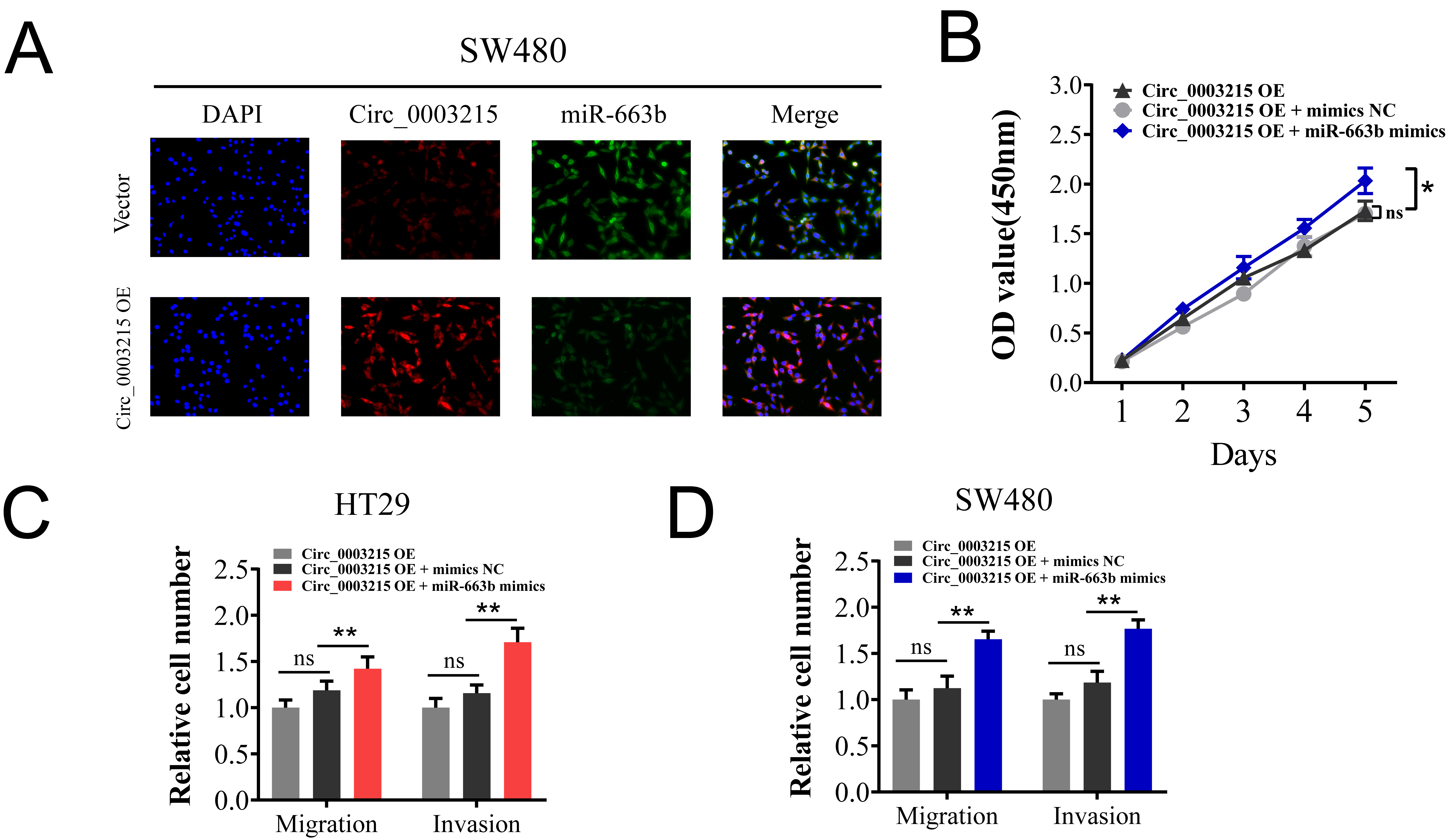

Supplement: Supplementary file 6 — Figure S4 [file 41419_2022_5245_MOESM6_ESM.png]

Fig.2

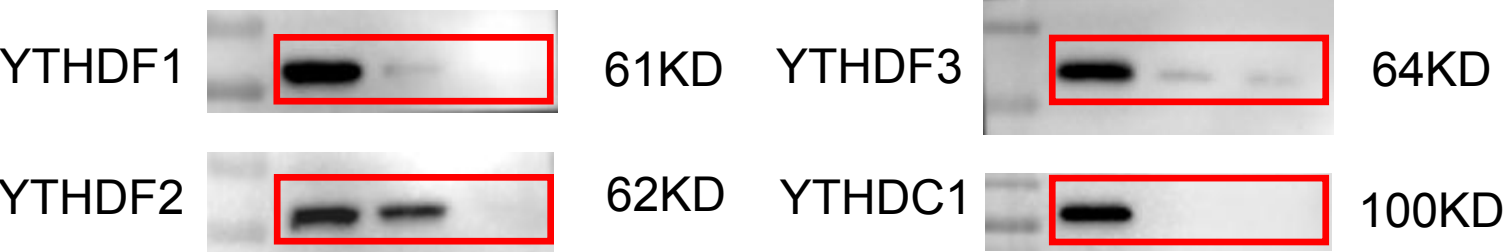

Fig.6

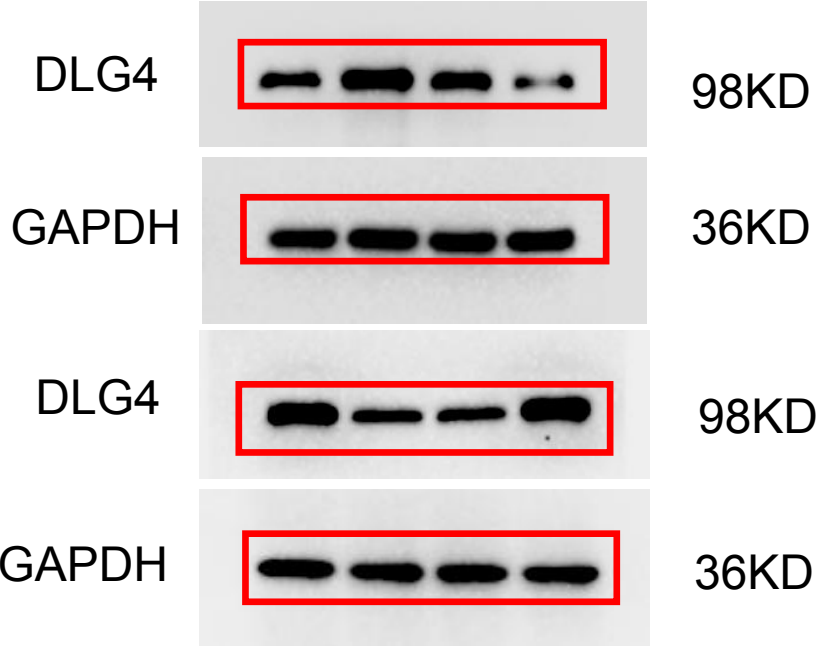

Fig.8C

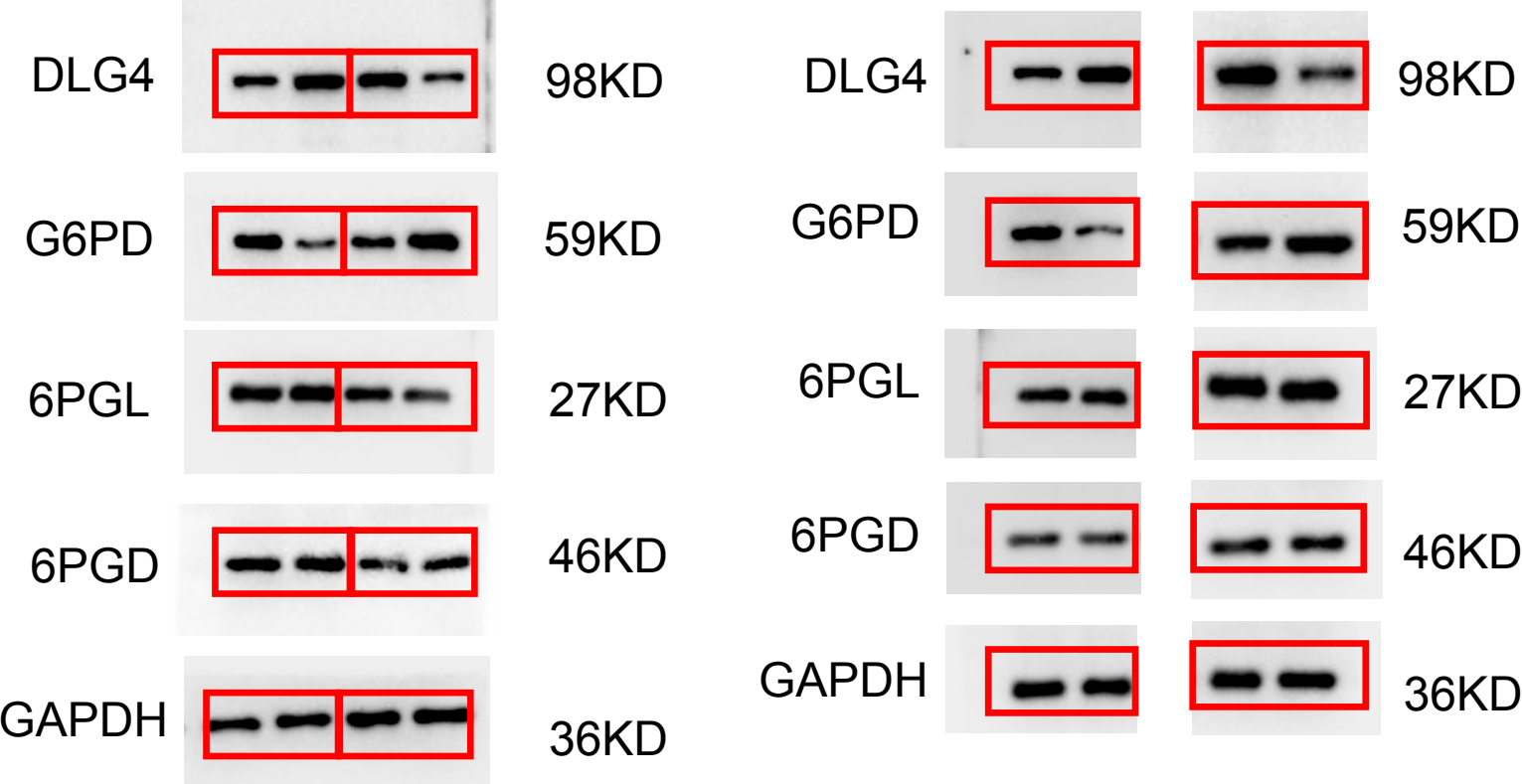

Fig.8C

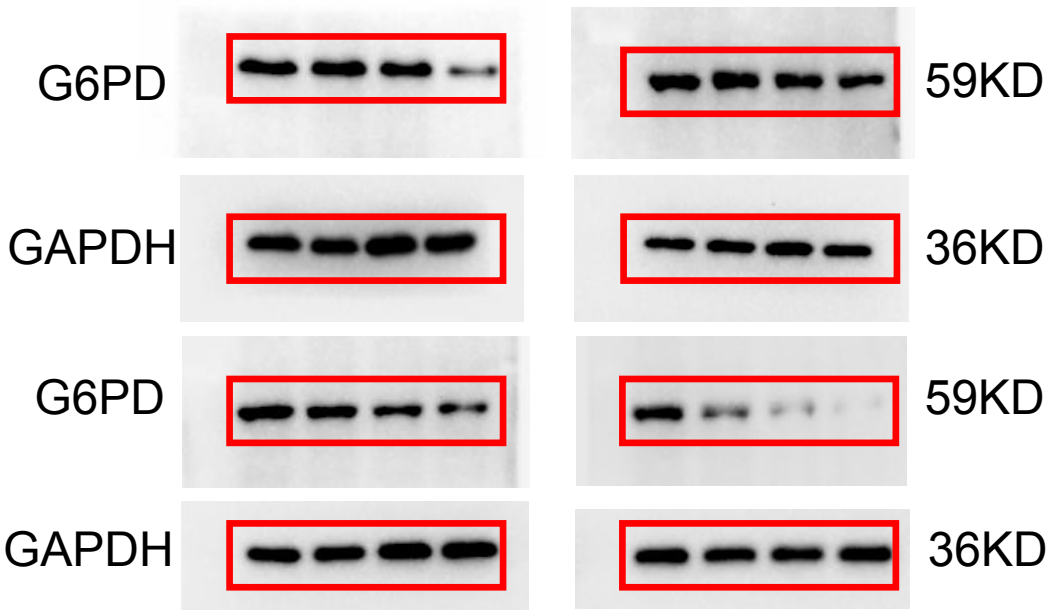

Fig.8D

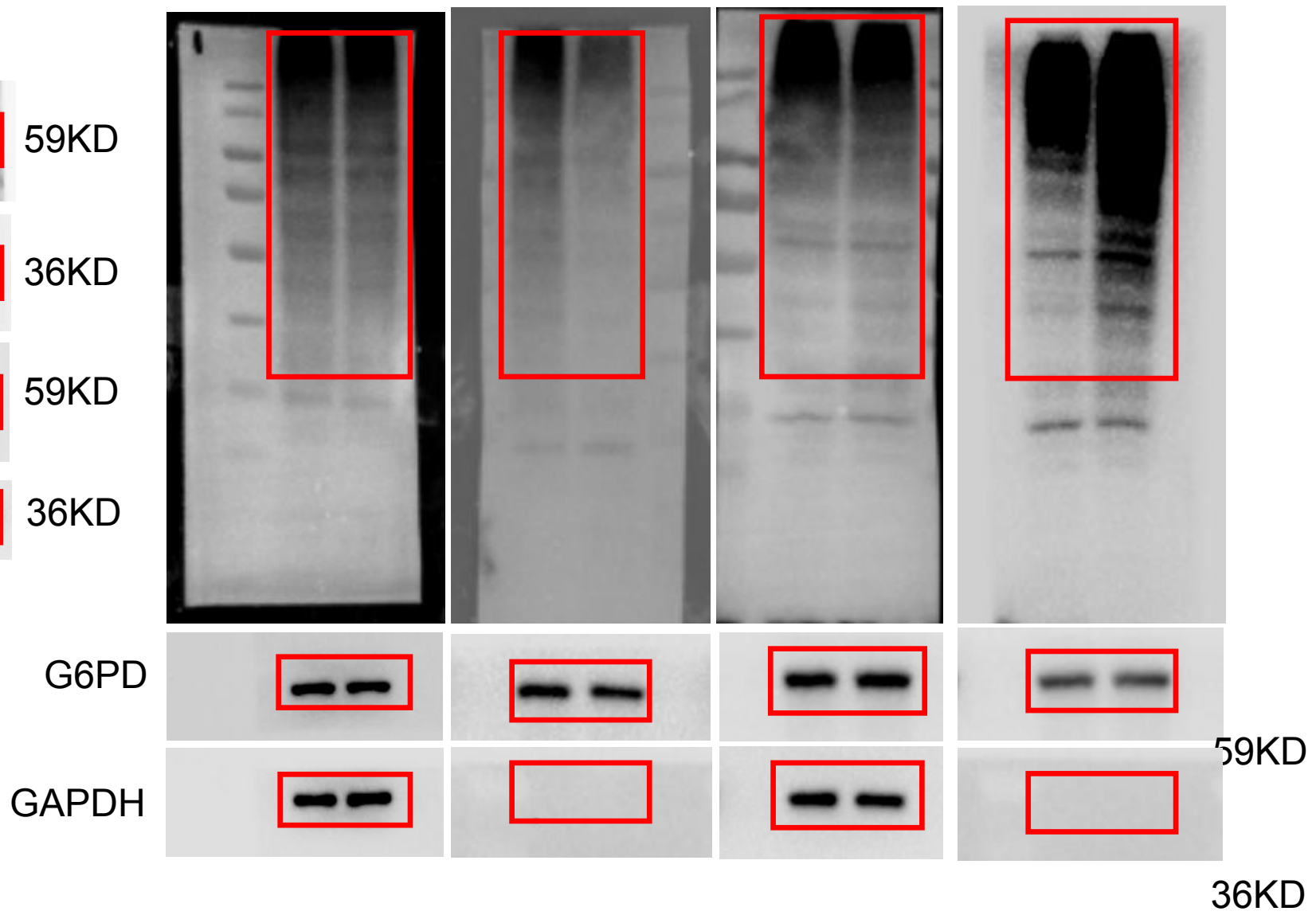

Fig.8F

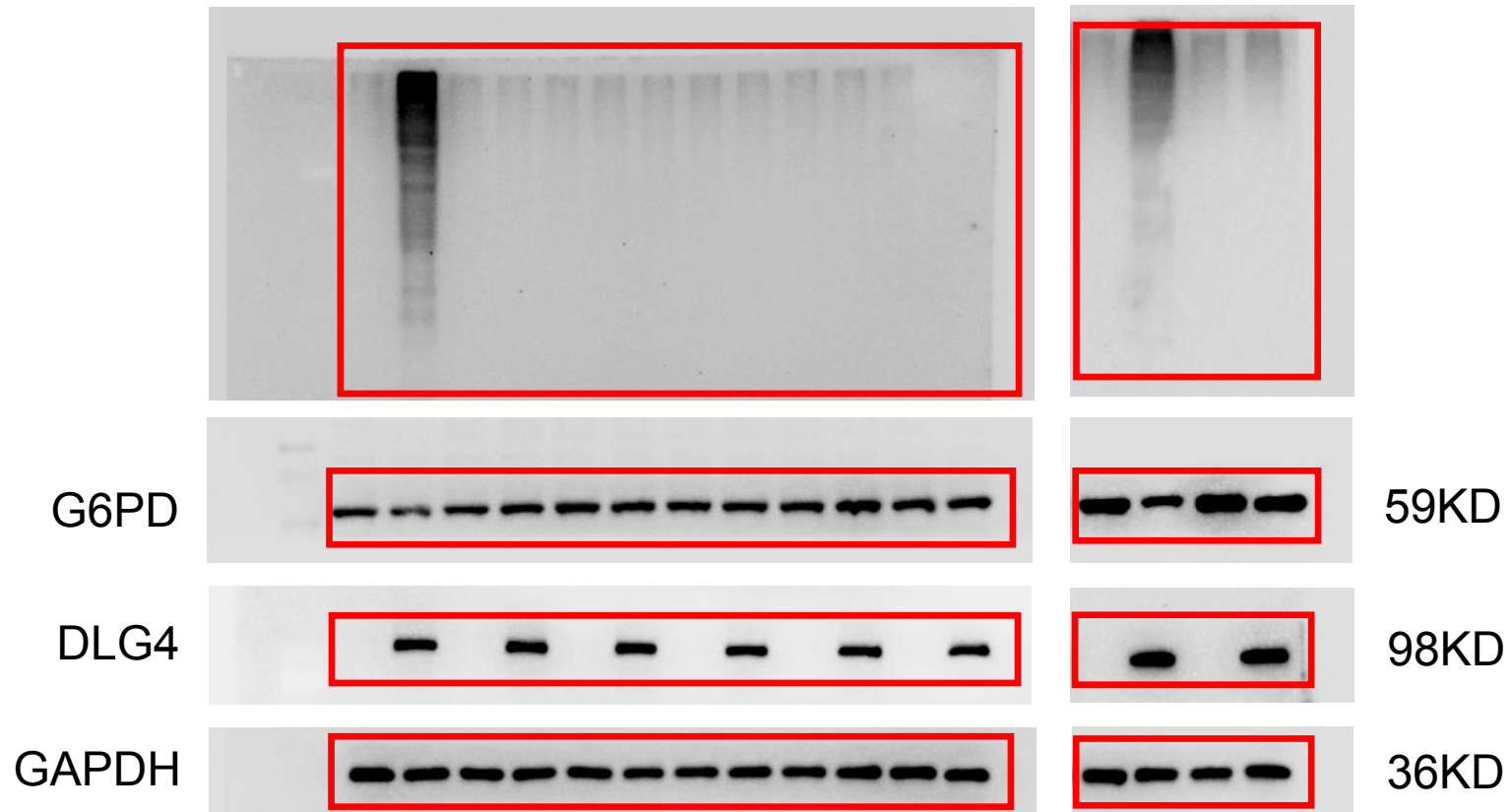

Fig.S5

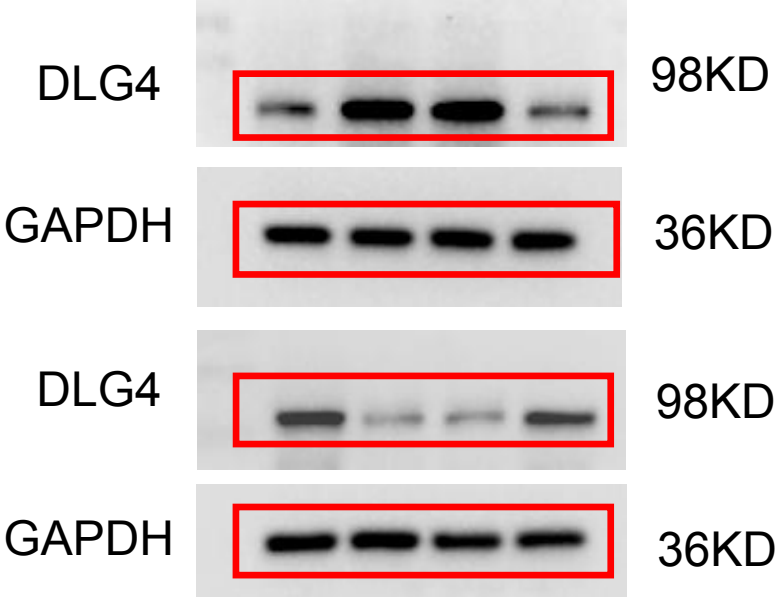

Fig.S6D

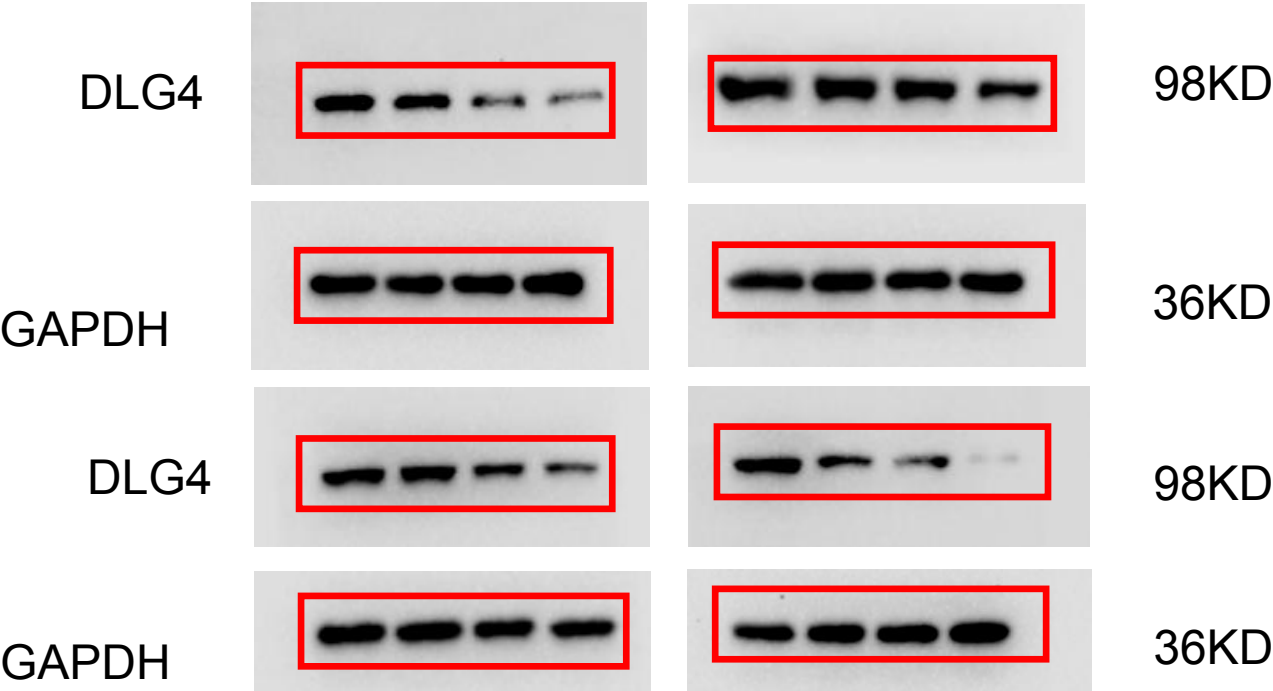

Fig.S6E

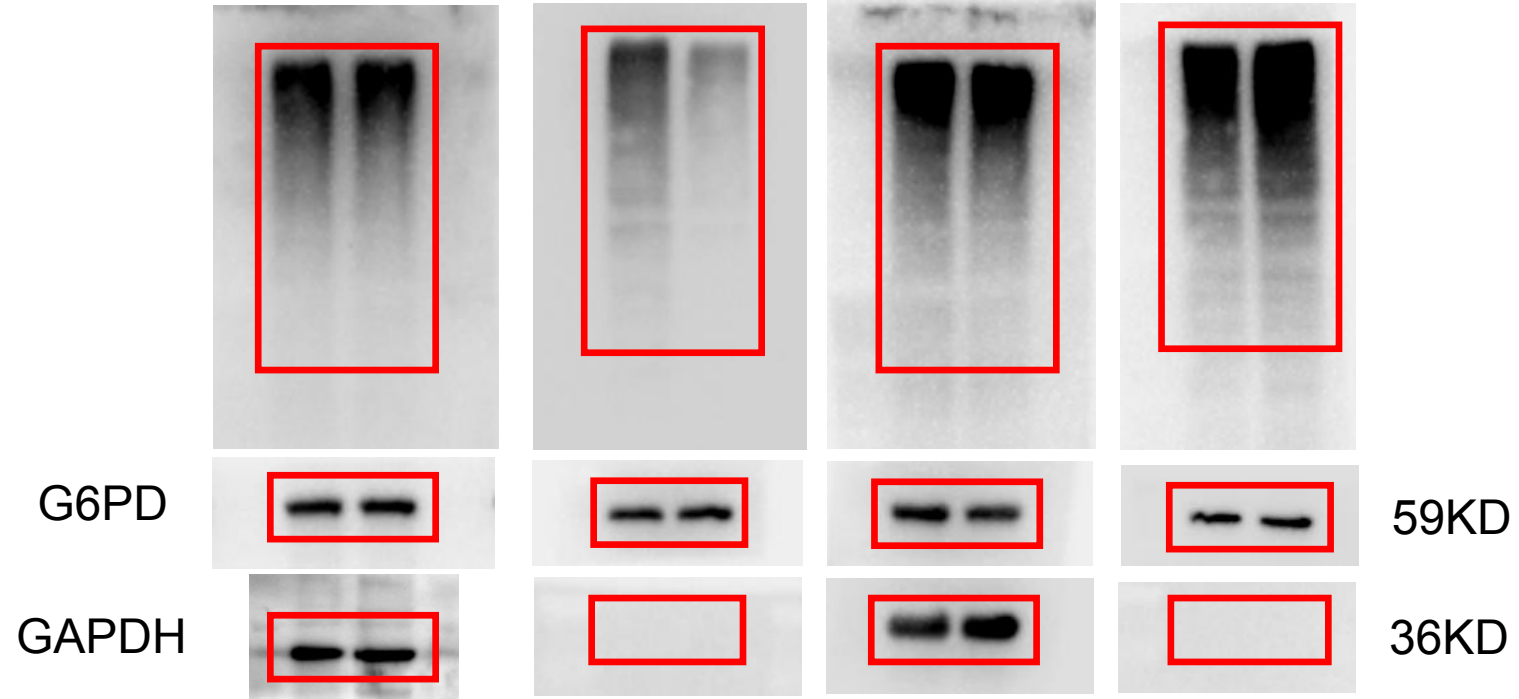

Supplement: Supplementary file 9 — Original Data File [file 41419_2022_5245_MOESM9_ESM.pdf]
