# Supplementary figures and images for: Correction: N6-methyladenosine modification of circ_0003215 suppresses the pentose phosphate pathway and malignancy of colorectal cancer through the miR-663b/DLG4/G6PD axis
Source: Cell Death Dis. 2025 Dec 23;16(1):909. doi: 10.1038/s41419-025-08199-3 (PMC12727862; doi:10.1038/s41419-025-08199-3)

Fig.2

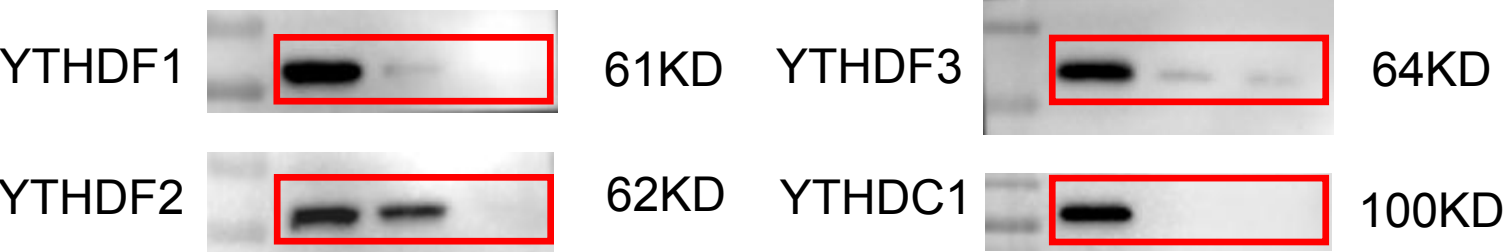

Fig.6

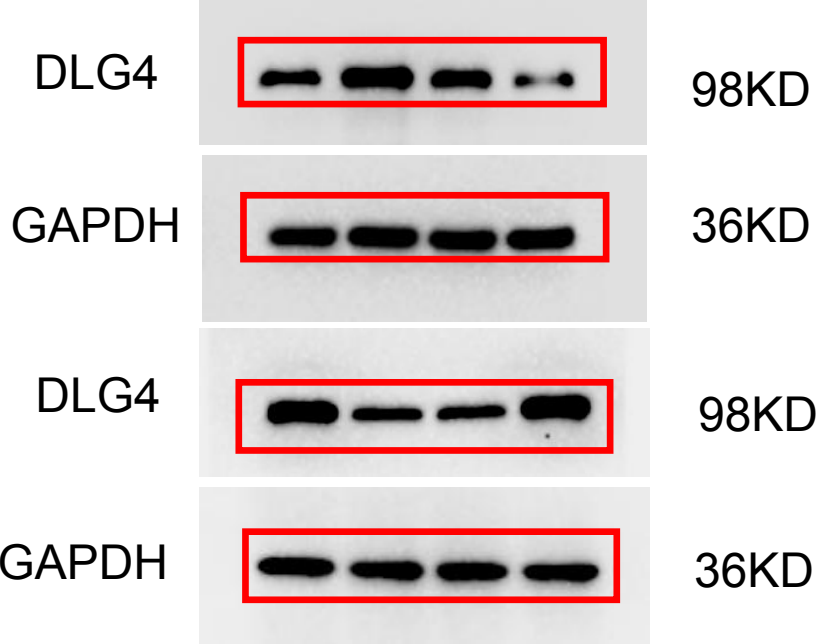

Fig.8C

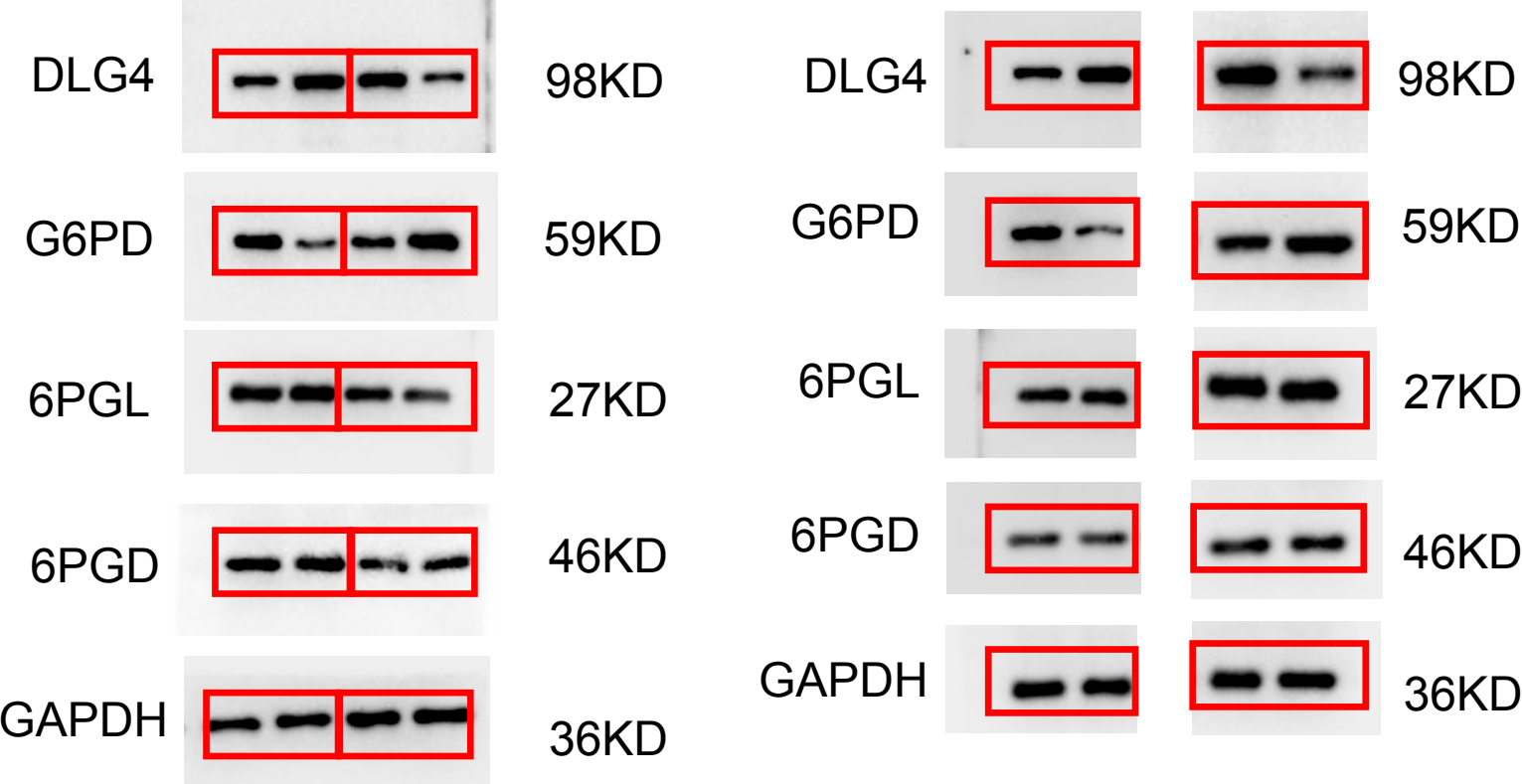

Fig.8C

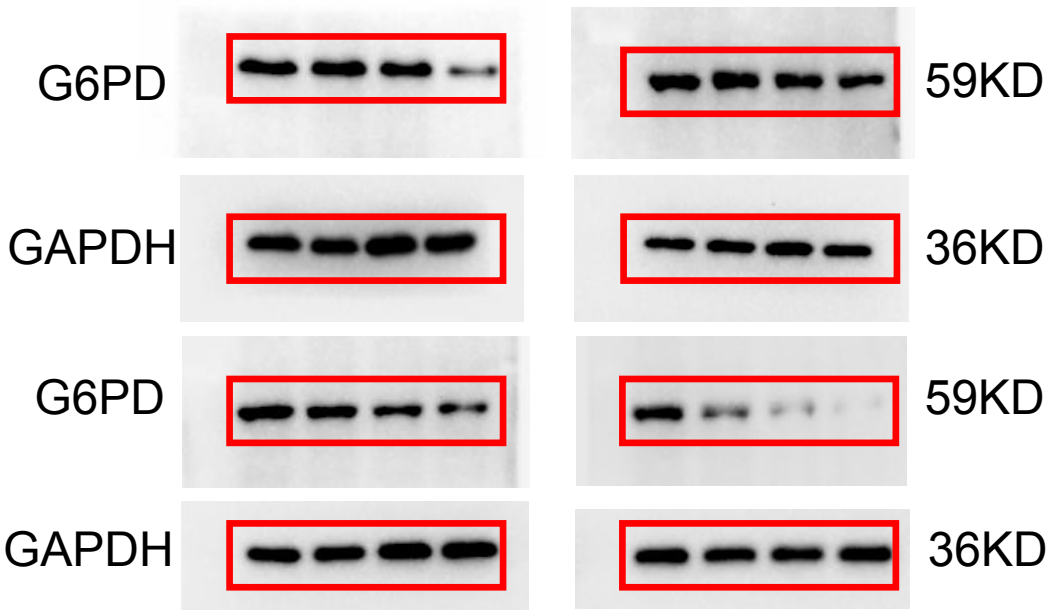

Fig.8D

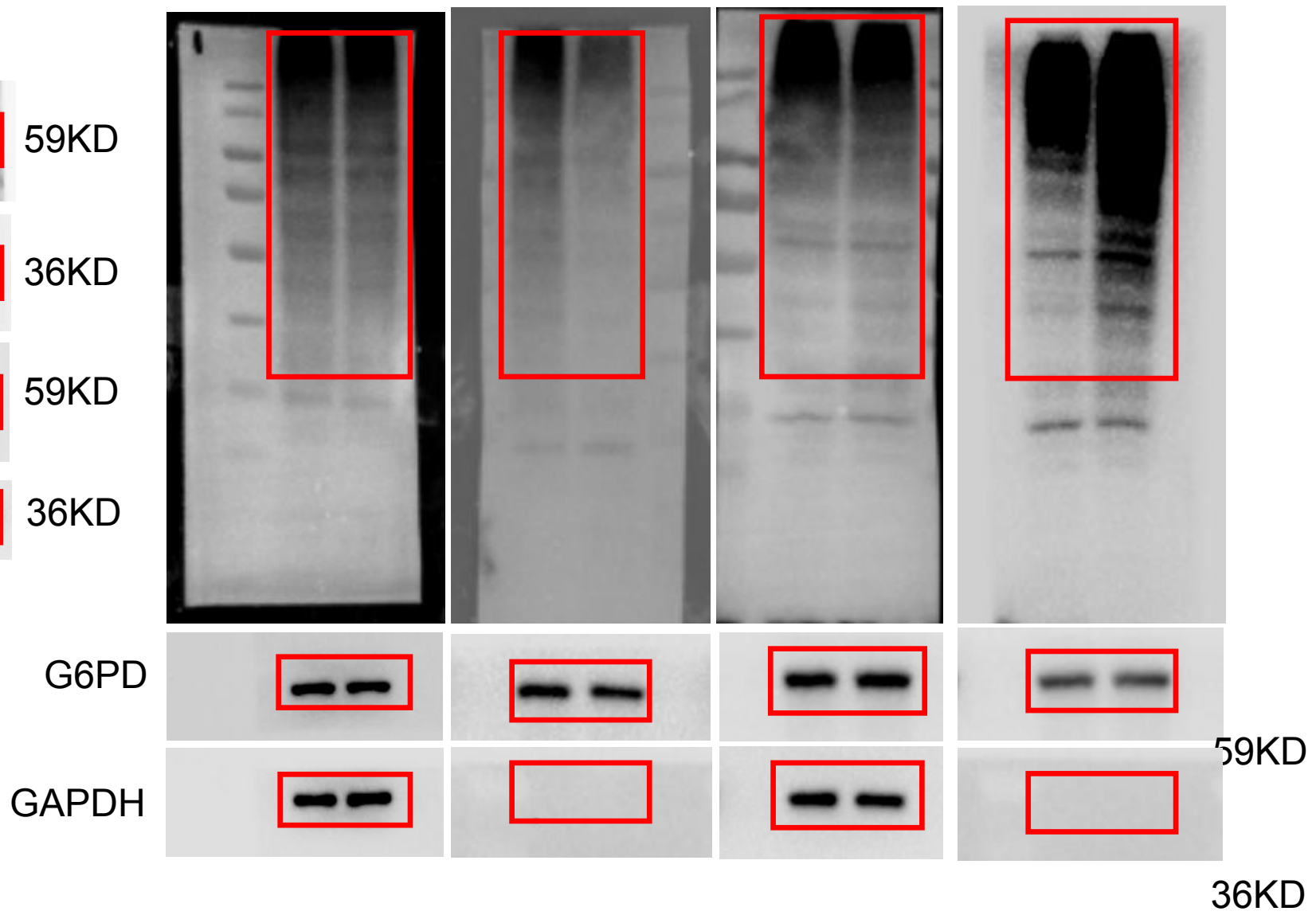

Fig.8F

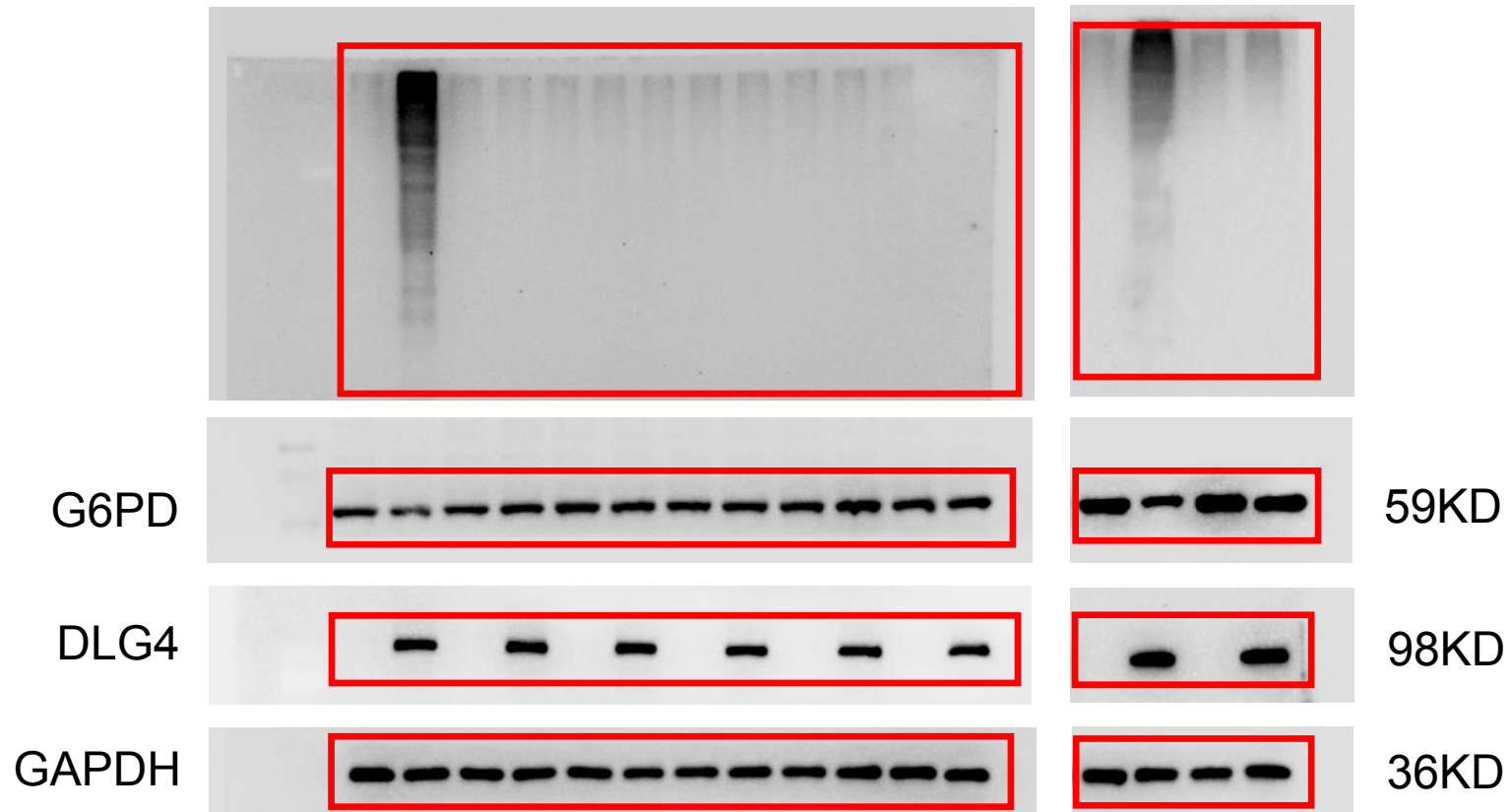

Fig.S5

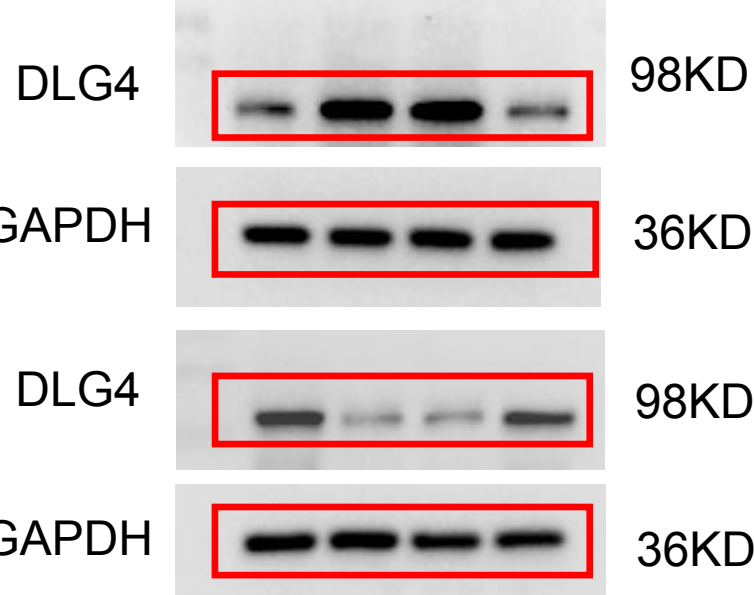

Fig.S6D

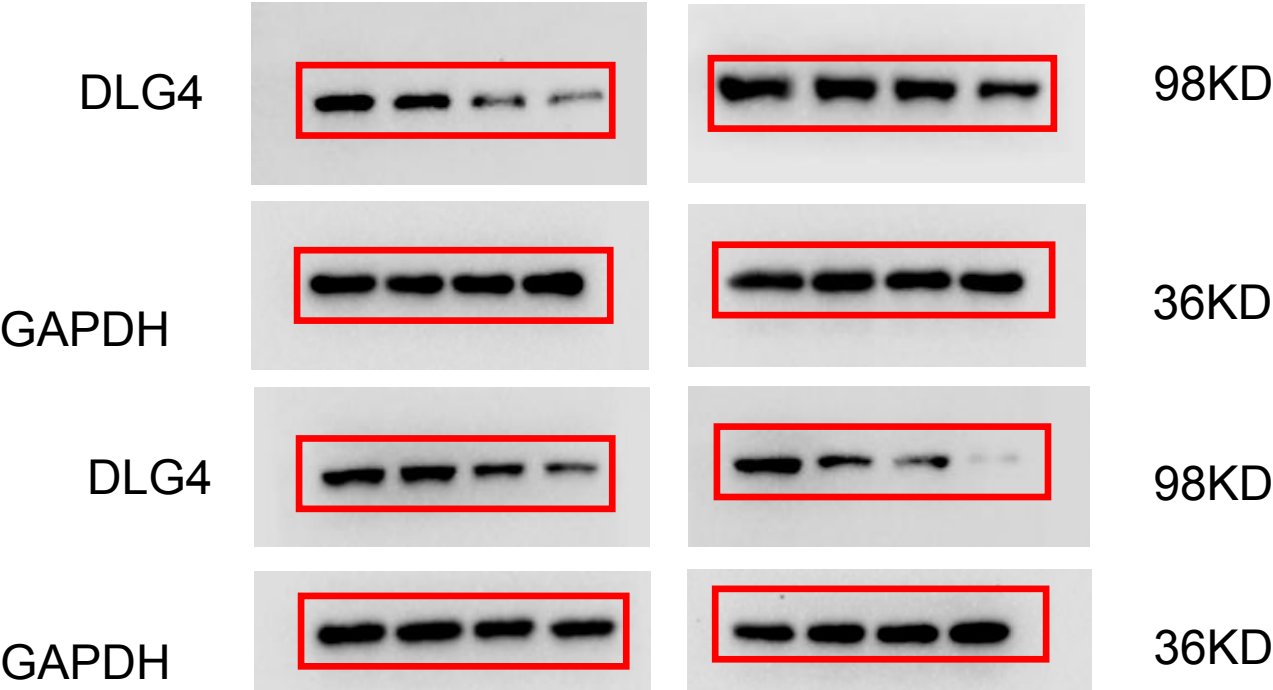

Fig.S6E

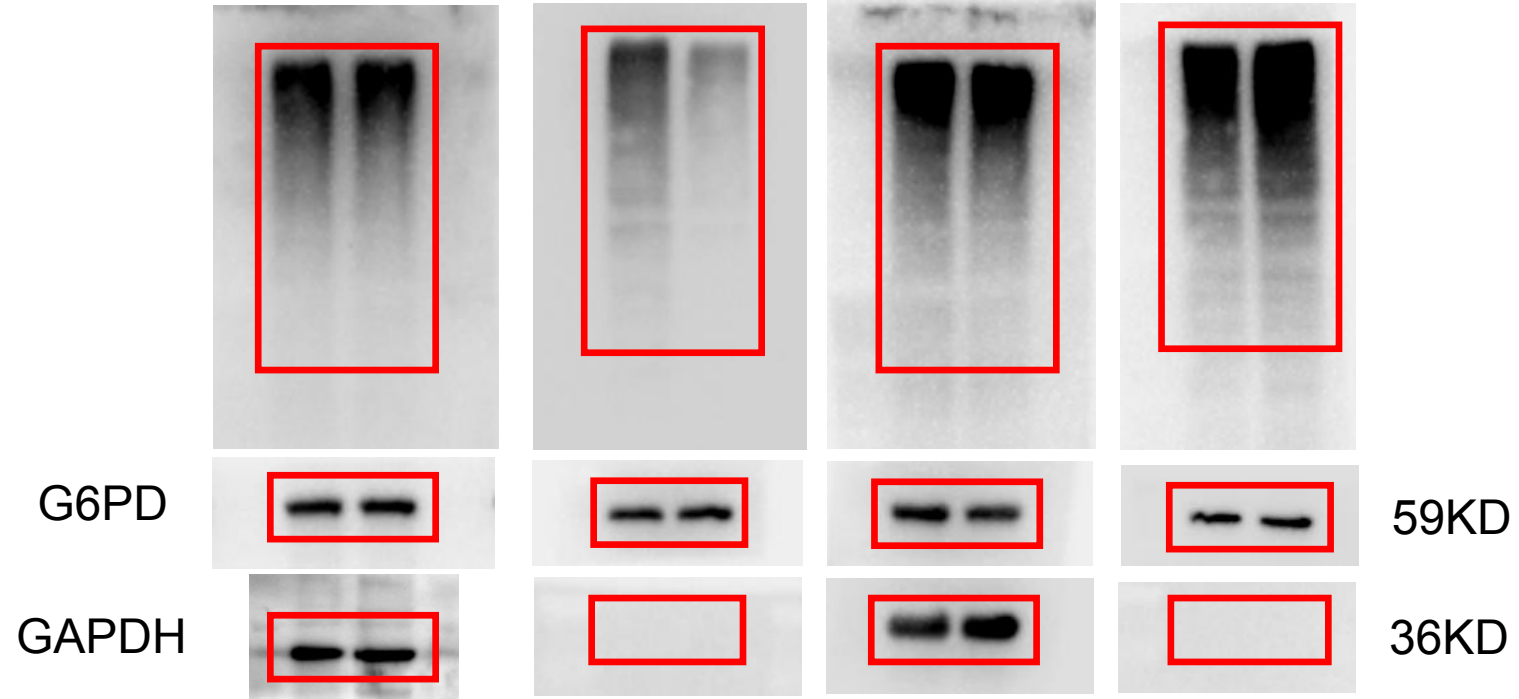

Supplement: Supplementary file 1 — Supplementary Original files [file 41419_2025_8199_MOESM1_ESM.pdf]
